# Assessment of IgG3 as a serological exposure marker for *Plasmodium vivax* in areas with moderate–high malaria transmission intensity

**DOI:** 10.3389/fcimb.2022.950909

**Published:** 2022-08-09

**Authors:** Yanie Tayipto, Jason Rosado, Dionicia Gamboa, Michael T. White, Benson Kiniboro, Julie Healer, D. Herbert Opi, James G. Beeson, Eizo Takashima, Takafumi Tsuboi, Matthias Harbers, Leanne Robinson, Ivo Mueller, Rhea J. Longley

**Affiliations:** ^1^ Population Health and Immunity Division, Walter and Eliza Hall Institute of Medical Research, Melbourne, VIC, Australia; ^2^ Department of Medical Biology, University of Melbourne, Melbourne, VIC, Australia; ^3^ Unité Malaria: Parasites et Hôtes, Département Parasites et Insectes Vecteurs, Institut Pasteur, Paris, France; ^4^ Laboratorio International Centers of Excellence for Malaria Research (ICEMR)-Amazonia, Laboratorios de Investigación y Desarrollo, Facultad de Ciencias y Filosofía, Universidad Peruana Cayetano Heredia, Lima, Peru; ^5^ Vector Borne Disease Unit, Papua New Guinea Institute of Medical Research, Goroka, Papua New Guinea; ^6^ Life Sciences, Burnet Institute, Melbourne, VIC, Australia; ^7^ Department of Immunology and Pathology, Monash University, Melbourne, VIC, Australia; ^8^ Department of Medicine, The Doherty Institute, The University of Melbourne, Melbourne, VIC, Australia; ^9^ Department of Microbiology and Central Clinical School, Monash University, Clayton, VIC, Australia; ^10^ Proteo-Science Center, Ehime University, Matsuyama, Japan; ^11^ CellFree Sciences Co., Ltd., Yokohama, Japan; ^12^ RIKEN Centre for Integrative Medical Sciences, Yokohama, Japan

**Keywords:** malaria, *Plasmodium vivax*, multiplex assay, surveillance, malaria elimination, antibody, IgG3

## Abstract

A more sensitive surveillance tool is needed to identify *Plasmodium vivax* infections for treatment and to accelerate malaria elimination efforts. To address this challenge, our laboratory has developed an eight-antigen panel that detects total IgG as serological markers of *P. vivax* exposure within the prior 9 months. The value of these markers has been established for use in areas with low transmission. In moderate–high transmission areas, there is evidence that total IgG is more long-lived than in areas with low transmission, resulting in poorer performance of these markers in these settings. Antibodies that are shorter-lived may be better markers of recent infection for use in moderate–high transmission areas. Using a multiplex assay, the antibody temporal kinetics of total IgG, IgG1, IgG3, and IgM against 29 P*. vivax* antigens were measured over 36 weeks following asymptomatic *P. vivax* infection in Papua New Guinean children (*n* = 31), from an area with moderate–high transmission intensity. IgG3 declined faster to background than total IgG, IgG1, and IgM. Based on these kinetics, IgG3 performance was then assessed for classifying recent exposure in a cohort of Peruvian individuals (*n* = 590; age 3–85 years) from an area of moderate transmission intensity. Using antibody responses against individual antigens, the highest performance of IgG3 in classifying recent *P. vivax* infections in the prior 9 months was to one of the Pv-fam-a proteins assessed (PVX_125728) (AUC = 0.764). Surprisingly, total IgG was overall a better marker of recent *P. vivax* infection, with the highest individual classification performance to RBP2b_1986-2653_ (PVX_094255) (AUC = 0.838). To understand the acquisition of IgG3 in this Peruvian cohort, relevant epidemiological factors were explored using a regression model. IgG3 levels were positively associated with increasing age, living in an area with (relatively) higher transmission intensity, and having three or more PCR-detected blood-stage *P. vivax* infections within the prior 13 months. Overall, we found that IgG3 did not have high accuracy for detecting recent exposure to *P. vivax* in the Peruvian cohort, with our data suggesting that this is due to the high levels of prior exposure required to acquire high IgG3 antibody levels.

## Introduction

*Plasmodium vivax* is a malaria-causing parasite that is mostly found outside sub-Saharan Africa. Like other *Plasmodium* species, *P. vivax* is transmitted by female *Anopheles* mosquitoes. Infected mosquitoes inject saliva containing sporozoites into the skin during feeding times. Sporozoites travel to the liver where some become arrested, a stage known as hypnozoites, and others develop into merozoites that are released into the bloodstream. Merozoites then infect red blood cells, while hypnozoites can later reactivate and develop into merozoites, resulting in a relapse of infection. This biological characteristic of *P. vivax* presents challenges for malaria control programs. Hypnozoites hidden in the liver are not detected by current diagnostic tests (including microscopy, rapid diagnostic tests, and PCR), as they can only detect parasites present in the blood. Current diagnostics tests also have low sensitivity, largely because the density of blood-stage *P. vivax* parasites is low due to the preference of *P. vivax* for young red blood cells that are more abundant in other organs, such as the spleen (Jiménez et al., 2007; Lacerda et al., 2012; Aggarwal et al., 2014) and bone marrow (O'Donnell et al., 1998; Imirzalioglu et al., 2006; Ru et al., 2009; Raghunandan et al., 2012).

To improve screening tools for malaria and to accelerate toward elimination, our laboratory has developed a serological tool that utilizes antibodies to infer recent exposure history to *P. vivax*. This tool can predict the likelihood of *P. vivax* infection within the prior 9 months in low transmission areas, such as Thailand, Brazil, and the Solomon Islands (Longley et al., 2020). People identified as exposed, but who have not received antimalarial treatment, could then be administered with radical cure (complete elimination of parasites) for *P. vivax*, which by necessity includes primaquine or tafenoquine to clear liver-stage hypnozoites. This provides a unique opportunity for an alternative strategy to rapidly reduce *P. vivax* transmission within a selected area and accelerate elimination efforts. This approach, termed sero-testing and treatment (“seroTAT”), has potential advantages over other strategies for eliminating the hypnozoite reservoir, such as mass testing and treatment and mass drug administration (Greenhouse et al., 2019). Mass testing and treatment will not efficiently eliminate the infectious reservoir due to the low sensitivity of current commercial rapid diagnostic tests and their reliance on detecting parasites in the peripheral blood (i.e., not hypnozoites (Sutanto et al., 2018)). Even if sensitive molecular tools such as PCR were used, they only detect individuals with current blood-stage infections and not those with hidden hypnozoites. Mass drug administration, conversely, is effective at reducing *P. vivax* transmission (Hsiang et al., 2013) but results in substantial overtreatment in low transmission settings which is potentially challenging due to i) potential side effects of 8-aminoquinolines in individuals with glucose-6-phosphate-dehydrogenase deficiency (Ashley et al., 2014), ii) lack of hypnozoite-clearing antimalarials approved for use in people who are pregnant, and iii) community acceptability (Aung et al., 2021).

Further studies conducted by our laboratory, however, have indicated that the performance of our serological tool was poorer in a Peruvian cohort from an area with moderate transmission intensity (during the last 9 months of follow-up, 65.6% of individuals experienced at least one blood-stage *P. vivax* infection) (Rosado et al., 2021). IgG responses to the target antigens appear to be longer-lived in this population due to repeated exposures. This is supported by prior evidence demonstrating longer-lived antibodies in higher compared to lower transmission settings (Longley et al., 2017). Hence, we aimed to adapt our tool for areas with moderate–high transmission intensity by using a different type of antibody as a marker of exposure. *P. vivax* infections typically induce cytophilic IgG1 and IgG3 subclass responses, with minimal IgG2 and IgG4 (Liu et al., 2022). We hypothesized that a shorter-lived serological response would be a better marker of exposure than total IgG (Tayipto et al., 2022). For example, a study in Uganda indicated that among IgG subclasses, IgG3 to most *Plasmodium falciparum* blood-stage antigens waned when transmission moved from high to low (Ssewanyana et al., 2021). Thus, in this study, we aimed to characterize the longevity of a panel of antibody isotypes and subclasses, including IgG3, following *P. vivax* infection, and then to test the response that was shorter-lived for its ability to act as a marker of recent *P. vivax* exposure in an area with moderate transmission intensity.

## Materials and methods

### Study populations

#### Papua New Guinea cohort: Antibody kinetics

**Figure 1:**
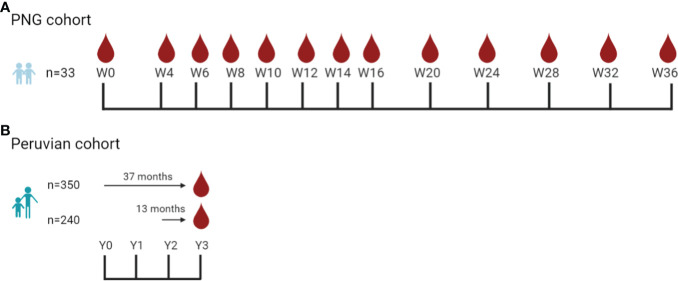
Sample collection timepoints used in the antibody kinetics study and the classification assessment study. **(A)** Asymptomatic children aged 5–10 years (*n* = 31) were enrolled in the Papua New Guinea (PNG) cohort study (Albinama), conducted in Maprik District, PNG, from August 2009 to May 2010 (Robinson et al., 2015). There were no reinfections during follow-up. Samples were collected at 13 timepoints for 36 weeks. **(B)** The Peruvian cohort study was conducted in Cahuide and San José de Lupuna, Loreto, Peru, from December 2012 to December 2015 (Rosas-Aguirre et al., 2017). Serum collected at the end of the study in the Peruvian cohort (Rosas-Aguirre et al., 2017) was used to assess the performance of IgG3 and total IgG antibody levels as markers for classifying recent *P. vivax* infection in the prior 9 months. In total, there were 590 individuals aged 3–85 years: 350 samples from individuals that were followed up for 37 months and 240 samples from individuals that were followed up for 13 months and qPCR data available in the last 13 months. Created in BioRender.com.

The Papua New Guinea (PNG) cohort study was conducted in Maprik District, East Sepik Province, PNG, from August 2009 to May 2010, where *P. vivax* was hyperendemic (Robinson et al., 2015). Malaria transmission in the province is moderately seasonal, with a peak from December to March in line with the wet season. Five hundred and twenty-four children aged 5–10 years were enrolled in this study regardless of *Plasmodium* infection status at the time of enrolment (subsequently, 47.4% were shown to be *P. vivax* positive at enrolment *via* PCR). Participants were randomized to receive the following antimalarial treatments to clear infections: chloroquine and artemether–lumefantrine for 3 days and either a placebo or primaquine for 20 days. For this project, samples from a subset of 31 children were selected as described previously (Longley et al., 2017). Briefly, these children had asymptomatic *P. vivax* infection at enrolment, had no *Plasmodium* reinfection during follow-up (with not more than one missed sample), and were randomized to receive primaquine (an anti-hypnozoite drug, all 31 children received primaquine treatment). Only the children that fulfilled these criteria were selected, leading to the final sample size of 31. Samples were collected up to 36 weeks: at enrolment, 1 month after enrolment (once drug treatment was completed), then every 2 weeks for 12 weeks, and every 4 weeks for the remaining weeks (**Figure 1A**). The number of samples available for each timepoint was as follows: week 0 (*n* = 31), week 4 (*n* = 29), week 6 (*n* = 31), week 8 (*n* = 30), week 10 (*n* = 27), week 12 (*n* = 29), week 14 (*n* = 30), week 16 (*n* = 30), week 20 (*n* = 25), week 24 (*n* = 22), week 28 (*n* = 28), week 32 (*n* = 25), and week 36 (*n* = 31).

#### Peruvian cohort: Classification performance

The Peruvian observational cohort study was conducted in Cahuide and San José de Lupuna, Loreto, Peru, from December 2012 to December 2015 (Rosas-Aguirre et al., 2017). Transmission at that time was stable in both Lupuna and Cahuide, with a peak season from November to May. There were 2,197 participants (from a possible 2,447 according to census data) scheduled for enrolment and follow-up in this cohort to enable estimates of population-based incidence rates of malaria in two different ecological settings (Cahuide: riverine; Lupuna: road-associated deforestation), with the goal of better understanding temporal and spatial dynamics of malaria transmission (Rosas-Aguirre et al., 2021). Initially, 1,029 participants were enrolled and followed with passive case detection monthly for 12 months, with a subsample of 456 individuals, then followed up monthly for another 24 months. Participants detected with microscopically confirmed *P. vivax* were treated with chloroquine for 3 days and primaquine for 7 days. The PCR prevalence at the beginning of the cohort was 16% for *P. vivax* and 2% for *P. falciparum* (Rosado et al., 2021 *).* Serum samples analyzed in this project were collected at the end of the study period (*n* = 590, aged 3–85 years) and had qPCR data from at least the prior 13 months. This included 350 participants that were followed up for 37 months and another 240 participants that were followed up for 13 months (Rosado et al., 2021) (**Figure 1B**). In the final year of follow-up, December 2014 to December 2015, a total of 7,612 blood samples were collected with 14.2% (1,083/7,612) of these positive for *P. vivax* by PCR (Rosado et al., 2020). Of these *P. vivax* PCR-positive samples, only 11.8% (128/1,083) were positive by microscopy and only 2.8% were symptomatic (30/1,083) (Rosado et al., 2020).

#### Negative controls

Plasma samples for the negative control panel were collected from 102 volunteers from the Volunteer Blood Donor Registry in Melbourne (VBDR), Australia; 100 volunteers from the Australian Red Cross (ARC) in Melbourne, Australia; and 72 samples from the Thai Red Cross (TRC), Bangkok, Thailand. These volunteers were unlikely to have had prior malaria infections as they were sourced from non-malaria endemic areas or countries. The VBDR excludes individuals who had a travel history in malaria-endemic areas. The TRC excludes individuals who have had a malaria infection in the last 3 years or traveled to malaria-endemic areas in the last year.

#### Informed consent and ethical approvals

All individuals gave informed consent and/or assent to participate in the study. The PNG cohort was approved by the PNG Institute of Medical Research Institutional Review Board (0908), the PNG Medical Advisory Committee (09.11), the Ethics Committee of Basel (237/11), and the WEHI HREC (approval numbers 14/02 and 07/07). The Peruvian cohort was approved by the Ethics Review Board of Universidad Peruana Cayetano Heredia (SIDISI code # 57395), the University of California San Diego Human Subjects Protection Program (Project # 100765), and the WEHI HREC (approval numbers 14/02 and 07/07). The use of the negative controls was approved by the WEHI (#14/02).

### Antigen coupling to magnetic beads

There were 32 P*. vivax* antigens used in this study (**Supplementary Table S1**). They were selected as potential markers of recent exposure to *P. vivax* based on data from previous studies (Longley et al., 2020). Twenty-eight proteins were made by CellFree Sciences Co., Ltd. (CFS) using the wheat germ cell-free (WGCF) expression system as previously described (Longley et al., 2020); three proteins [CSP210, CSP247 (PVX_119355), and AMA-1 (Palo Alto sequence)] were made at the Burnet Institute using the Expi293 expression system as previously described (Drew et al., 2017; Kurtovic et al., 2019); one antigen (PVX_090240 CyRPA) was made at the WEHI using a Baculovirus expression system as previously described (Longley et al., 2020); and one antigen (PVX_094255 RBP2b_161-1009_) was made at Ehime University using the WGCF system as previously described (Bourke et al., 2022). Not all antibody responses were measured for all cohorts for all proteins, due to the availability of protein at that time. IgG1 and IgG3 responses were not measured against PVX_082735 (TRAP/SSP2), PVX_090240 (CyRPA), and PVX_119355 (CSP247) in any cohort. Total IgG responses were not measured for any cohort against PVX_090240 (CyRPA) and PVX_094255 (RBP2b_161-1009_).

*P. vivax* antigens were coupled to magnetic BioPlex COOH beads [Bio-Rad South Granville, Australia, 171506(*xxx – unique for each region*)] following the manufacturer’s instructions, with modifications as per previously published methods (Mazhari et al., 2020). Briefly, each antigen was coupled to a unique set of microspheres. Stock microspheres were sonicated in a water bath for 15 s then vortexed for 10 s. Two hundred microliters of microspheres were immobilized in a magnetic separator rack (Bio-Rad, 1614916) for 30–60 s. The supernatant was removed, and the microspheres were resuspended with 200 μl of MQ-H_2_O. The resuspension was vortexed for 20 s and placed in the magnetic separator again. The supernatant was then removed. The microspheres were resuspended with 100 mM of monobasic sodium phosphate, pH 6.2, and vortexed for 20 s. Twenty microliters of 50 mg/ml sulfo-N-hydroxysuccinimide (S-NHS) (Sigma, 56485) in MQ-H_2_O was then added to the suspension and vortexed gently for 10 s, followed by 20 μl of 50 mg/ml of N-ethyl-N′-(3-(dimethylamino)propyl)carbodiimide (EDC) (Sigma, 3449) in MQ-H_2_O again with gentle vortexing for 10 s. The mixture was incubated for 20 min in the dark at room temperature on a tube rotator. The microcentrifuge tubes were then placed in a magnetic separator for 30–60 s and the supernatant was removed. The microspheres were resuspended with 500 μl of 1× phosphate-buffered saline (PBS), pH 7.4, and vortexed for 20 s. This washing process was repeated one more time. The antigens in 1× PBS, pH 7.4, were then added, following the amounts stated in **Supplementary Table S1**. After the addition of the antigen, the mixture was incubated at room temperature for 2 h or at 4°C overnight on a tube rotator. Then, the mixture was washed 3× using 500 μl of 1× PBS-TBN, pH 7.4 [made in-house, PBS, 0.1% bovine serum albumin (BSA) (Sigma, A7906), 0.02% Tween-20, 0.05% azide] before final resuspension in 500 μl of 1× PBS-TBN, pH 7.4. The coupled beads were stored at 4°C in the dark.

### Multiplex antibody assays

Antibody responses were measured in plasma samples using the antigen-coupled microspheres prepared as previously described. Variations of the assay were used to measure total IgG (Mazhari et al., 2020), IgG1 and IgG3 subclasses (Liu et al., 2022), and IgM (Longley et al., 2021). Plasma or sera samples were diluted in PBT (made in-house, 1× PBS, 1% BSA, 0.05% Tween-20) with the following dilution: 1/200 for IgM assay, 1/100 for total IgG, and 1/50 for IgG1 and IgG3 assays. A two-fold serial dilution of the positive control pool (PNG hyperimmune plasma) diluted from 1/50 to 1/25,600 was included on each plate. Blanks (PBT and beads, no plasma) were included to measure the fluorescence background, prepared in triplicate. Fifty microliters of diluted plasma and 50 μl of bead mixture were added to each well of a 96-well Greiner Bio-One plate (Interpath, Heidelberg West, Australia, 655090). The antigen-coupled bead mixture was composed of 0.1 μl of each antigen-coupled bead per well. The mixture of plasma and antigen was incubated for 30 min in the dark on a plate shaker. After incubation, the plate was washed 3× with 100 μl of PBT using a plate washer. One hundred microliters of the relevant secondary antibody was then added per well, with the following dilutions: 1/400 in PBT for IgM (Jackson ImmunoResearch, 709-116-073), 1/100 in PBT for total IgG (Jackson ImmunoResearch, Pennsylvania, USA, 709-116-098), and 1/50 in PBT for the IgG subclasses (IgG1: SouthernBiotech, 9052-09, IgG3: SouthernBiotech, Alabama, USA, 9210-09). Plates were incubated for 15 min in the dark on a plate shaker and then washed 3× with 100 μl of PBT. Finally, for all assay variations, the mixture was resuspended with 80 μl of PBT and incubated for at least 5 min in the dark on a plate shaker. The plate was then read on a MAGPIX instrument (Luminex, Austin, USA). Data readout from the machines was in median fluorescence intensity (MFI). Results were then checked: quality control ensured bead number per well >15, blanks were <50 MFI, and standard curves for each antigen were consistent across plates.

### Data analyses

For multiplexed assays (total IgG, IgG1, IgG3, IgM), MFI was converted to relative antibody units (RAU) to normalize the values of different plates using the positive control standard curve. This was performed using a five-parameter logistic regression model in the R program established in a previous study (Franca et al., 2016). For visualization in the figures of antibody kinetics, the antigen-specific background (median of negative controls) was subtracted from the median antibody level at each timepoint, in an antigen-specific manner. Raw RAU data are shown in the **Supplementary Figures**. A seropositivity cutoff was set at the average of negative controls plus two times the standard deviation. Where appropriate, antibody data were log-transformed prior to statistical analysis, as detailed in the *Results* section. Locally estimated scatterplot smoothing (LOESS) for antibody kinetic graphs was fitted using RStudio 1.4.1106 (Boston, USA). Antibody classification performance was analyzed using receiver operator characteristic (ROC) curves in R as previously described (Longley et al., 2020). The ROC curves plot sensitivity (true-positive rate) and 1-specificity (false-positive rate) at different classification thresholds or cutoffs. The area under the curve (AUC) is an aggregate of performance using all possible classification thresholds. Multiple linear regression models were performed in STATA/SE 16.1 (Texas, USA) to explore the effect of various epidemiological factors on the acquisition of IgG3 antibody levels. Correlation analysis was performed using Spearman in R. Correlations with *r* values <0.3 were considered weak, 0.3–0.7 moderate, and >0.7 strong correlations.

## Results

### Antigen-specific antibody kinetics following asymptomatic *P. vivax* infections in PNG children

*P. vivax* antigen-specific antibody kinetics (*n* = max 32 antigens) over 36 weeks were first characterized following asymptomatic *P. vivax* infections in 31 PNG children (5–10 years) from Maprik, an area with moderate–high transmission intensity, with the goal of identifying antibody response pattern/s that would suggest suitability as markers of recent exposure to *P. vivax.* Antibody responses in a panel of 274 malaria-naive individuals from non-malaria endemic areas were used as controls. Baseline refers to the median antigen-specific antibody response of the negative controls. A seropositivity cutoff was calculated as the median of negative controls plus two times the standard deviation.

#### Total IgG responses against 30 *P. vivax* antigens

**Figure 2:**
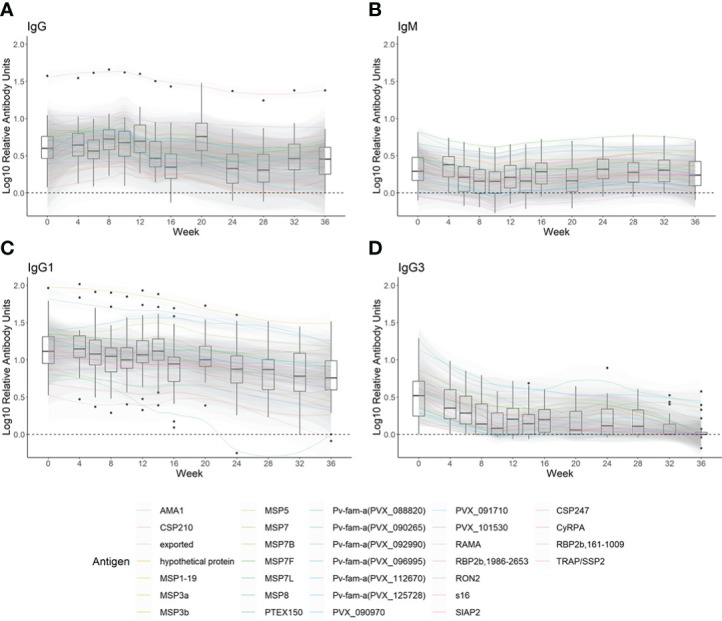
The kinetics of **(A)** total IgG, **(B)** IgM, **(C)** IgG1, and **(D)** IgG3 against 29–32 Plasmodium vivax antigens following asymptomatic P. vivax infections in PNG children. Antibody responses following asymptomatic P. vivax infections in PNG children (n = 31) over 36 weeks were measured using a multiplex assay. **(A)** Total IgG to 30 P. vivax antigens, **(B)** IgM to 32 P. vivax antigens, and **(C)** IgG1 and **(D)** IgG3 to 29 P. vivax antigens were measured. To standardize across antigens, the median of each antigen-specific antibody measured at each timepoint is shown after subtraction of the median antibody responses measured in negative controls (total IgG n = 274, IgM n = 260, IgG1 n = 248, and IgG3 n = 256). Box plots for each timepoint show the median of the adjusted medians of all antigen-specific antibodies, with black dots outside the whiskers indicating outlier protein responses. The kinetics of each type of antibody are presented in locally estimated scatterplot smoothing (LOESS) lines with 95% confidence intervals.

Total IgG levels in the PNG cohort to most antigens were above baseline and maintained for at least 12 weeks following asymptomatic *P. vivax* infection, followed by a slight decrease (**Figure 2A**). For most antigens, median IgG levels were maintained above baseline throughout the entire 36-week follow-up, with only a few exceptions including AMA1, the hypothetical protein (PVX_097715), and CSP210. RBP2b_1986-2653_ was highly immunogenic and a notable outlier with total IgG levels well above baseline over 36 weeks (**Figure 2A**). **Supplementary Figure S1** shows the total IgG antibody levels at a per-person level for each antigen separately and provides an indication of the antigen-specific seropositivity cutoff. Most individual IgG responses against RBP2b_1986-2653_ were also above the seropositivity cutoff for 36 weeks (**Supplementary Figure S1**). Total IgG levels against the other *P. vivax* antigens in PNG children often fell within the seropositivity cutoff. At the time of *P. vivax* infection (week 0), 3.0%–84.9% of individuals in the PNG cohort had a seropositive total IgG response (**Supplementary Table S2**). There were 10 proteins in this cohort where the total IgG level of the children, using a local regression line (LOESS), trended above the seropositivity cutoff at week 0 and declined over the 36 weeks (**Supplementary Figure S1**, indicated by an *).

#### IgM responses against 32 *P. vivax* antigens

IgM levels tended to be high at week 0 for most proteins (i.e., above baseline), with a slight decline over 10 weeks before rising again to the IgM level as detected at enrolment (**Figure 2B**). The IgM seropositivity at week 0 was 0%–75.8% (**Supplementary Table S2**). In contrast to the IgG responses in this cohort, there were fewer *P. vivax* antigens that elicited clear IgM responses following asymptomatic *P. vivax* infections in PNG children that were above the seropositivity cutoff (**Supplementary Figure S2**). Exceptions were for MSP7F and MSP7L where LOESS IgM levels were maintained slightly above the seropositivity cutoff for 36 weeks (**Supplementary Figure S2**). There were no antigens that induced the required IgM profile (for markers of recent exposure) of seropositive at week 0 followed by a decline in magnitude over 36 weeks.

#### IgG subclass responses against 29 *P. vivax* antigens

Median IgG1 levels to most *P. vivax* proteins were maintained, or declined slightly, over 36 weeks following asymptomatic *P. vivax* infection (**Figure 2C**). At the time of infection, IgG1 was detected in most *P. vivax* antigens (15.2%–97.0% seropositivity at week 0) (**Supplementary Table S2**). LOESS IgG1 levels to 14 P*. vivax* proteins were higher than the seropositivity cutoff at the time of infection, then declined to below the seropositivity cutoff within 36 weeks (**Supplementary Figure S3**, indicated by an *). This included 7/10 of the same antigens with this longevity pattern as per total IgG.

In comparison to IgG1, the decline in median IgG3 levels to most *P. vivax* antigens over time was more prominent, even reaching the baseline by 8 weeks post-infection to some antigens (**Figure 2D**). IgG3 was not induced as robustly as IgG1 at week 0 (seropositivity rates of 6.1%–69.7% per antigen) (**Supplementary Table S2**). LOESS IgG3 responses were elicited above the seropositivity cutoff at enrolment but then declined to below the seropositivity cutoff after 6–8 weeks to 18 out of 29 antigens (**Supplementary Figure S4**, indicated by an *). This included 6/10 of the same antigens with this longevity pattern as per total IgG, 10/14 as per IgG1, and 6 unique to IgG3. LOESS IgG3 levels to MSP3a were maintained above the seropositivity cutoff over 36 weeks (**Supplementary Figure S4**).

### Performance of IgG3 in classifying recent *P. vivax* infections in an area with moderate transmission intensity

Given the sharp decline in IgG3 levels over time to most *P. vivax* antigens in PNG following asymptomatic *P. vivax* infection, IgG3 was selected for further assessment as a potential serological exposure marker. Both the IgG3 response and total IgG (for comparison) to 29 P*. vivax* proteins were measured in samples collected in Cahuide and San José de Lupuna, Loreto, Peru (Rosas-Aguirre et al., 2017). The plasma samples used were collected at the end of a longitudinal cohort (*n* = 590) with qPCR data available from the previous 13 months (**Figure 1B**). A summary of the epidemiological characteristics of these samples is listed in **Supplementary Table S3**.

**Figure 3:**
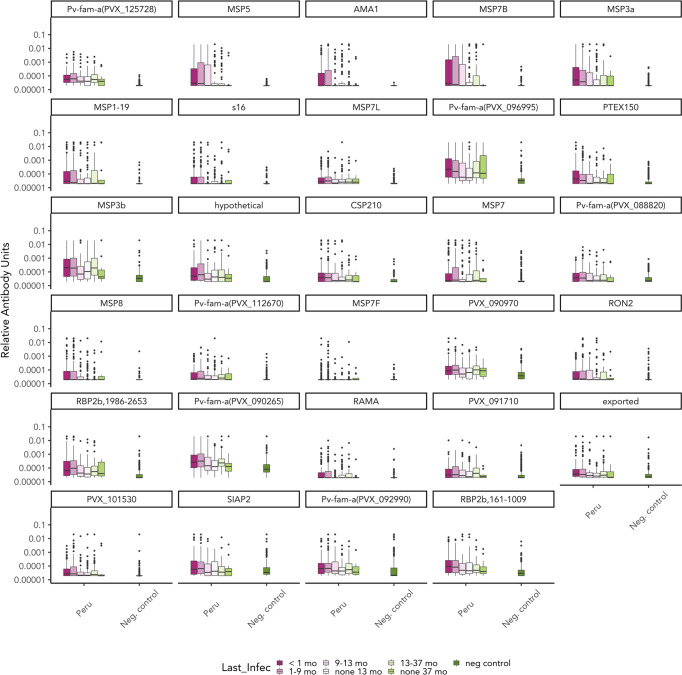
IgG3 responses to 29 Plasmodium vivax antigens of the Peruvian cohort based on time since prior P. vivax blood-stage infection. IgG3 responses to 29 P. vivax antigens were measured at the end of the Peruvian cohort (n = 590). IgG3 antibody responses are presented in relative antibody units (log) with box plots showing the median with interquartile range. The level of antibody was classified based on the prior P. vivax infection status as detected by qPCR: currently infected (<1 month) (n = 160), infected in the last 1–9 months (n = 228), infected in the last 9–13 months (n = 56), not infected within the last 13 months (n = 75), infected in the last 13–37 months (n = 59), and not infected in the last 37 months (n = 12). Antigens are ordered by the highest seropositivity rates (see **Table S4**).

IgG3 responses to most *P. vivax* antigens were low (**Figure 3**). The seropositivity rate of IgG3 was less than 20% to 21 out of 29 P*. vivax* antigens in the panel (calculated on those infected within the last 9 months, **Supplementary Table S4**). Only one of the Pv-fam-a proteins (PVX_125728) had IgG3 seropositivity >50% in the overall cohort and among those infected in the last 9 months. In addition, there was a high level of individual variability in the IgG3 response generated as indicated by outlier data points, such as to Pvs16 (**Figure 3**). In comparison, total IgG levels to most *P. vivax* proteins were strongly induced in the Peruvian cohort (**Supplementary Figure S5**), with clear patterns of decreasing IgG with increasing time since prior infection to proteins such as MSP5 and AMA1. In total, 12 P*. vivax* proteins had significantly higher mean antibody levels in individuals with *P. vivax* infections in the prior 9 months compared to those with no infections or infections more than 9 months ago (**Supplementary Figure S5**). Notably, only three of these were antigens that also had a short-lived total IgG profile in the PNG children cohort (MSP7L, MSP3a, RBP2b_1986-2653_). There were six antigens where total IgG responses were detected at similar levels regardless of the recency of *P. vivax* infection, suggesting a long-lived IgG response. Notably, this included three antigens that had a short-lived profile in PNG children (MSP1-19, the Pv-fam-a protein PVX_125728, and MSP7B). The remainder was poorly immunogenic in the Peruvian individuals with *P. vivax* infections in the prior 9 months (<40% seropositivity, **Supplementary Table S4**). The seropositivity of total IgG to most antigens was higher than that of IgG3 in the Peruvian individuals with *P. vivax* infection history (**Supplementary Table S4**).

**Figure 4:**
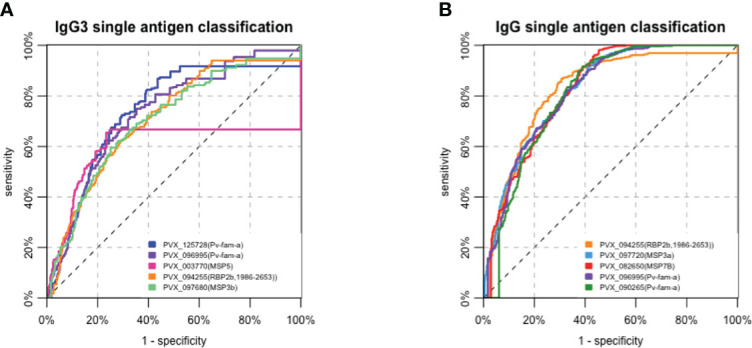
Receiver operating characteristic (ROC) curves of the top 5 **(A)** IgG3 and **(B)** total IgG responses to antigens in classifying recent Plasmodium vivax exposure (within 9 months) in a Peruvian cohort with moderate transmission intensity. The performance of each P. vivax antigen in the serological marker panel for classifying recent P. vivax infections in the prior 9 months was assessed individually using an ROC curve. **(A)** The top 5 antigen-specific IgG3 responses that can classify P. vivax infection were against the following: two Pf-vam-a proteins (PVX_125728, PVX_096995), MSP5, RBP2b1986-2653, and MSP3b (AUC value = 0.714–0.764). **(B)** The top 5 antigen-specific total IgG responses were against the following: RBP2b1986-2653, MSP3a, MSP7B, and two Pv-fam-a proteins (PVX_096995, PVX_090265) (AUC value = 0.818–0.833).

The classification performance of each antibody was then assessed, with qPCR data as reference for time since previous *P. vivax* infection. The balance of sensitivity and specificity when using varying antibody levels as the cutoff for each *P. vivax* protein is depicted in receiver operator characteristic (ROC) curves. The area under the ROC curve (AUC) value was used to summarize these results (no predictive power AUC = 0.5, perfect predictor AUC = 1). IgG3 resulted in poor performance compared to total IgG in classifying recent *P. vivax* infection within the previous 9 months. Only 6 out of 29 antigen-specific IgG3 responses resulted in AUC >0.7 (range 0.571–0.764, **Table 1**). In contrast, total IgG responses to most (26/29) *P.* vivax proteins had an AUC value of >0.7 (range 0.589–0.833) (**Supplementary Table S5**). The top 5 antigens that were able to classify recent *P. vivax* infection using IgG3 were two Pv-fam-a proteins (PVX_125728, PVX_096995), MSP5, RBP2b_1986-2653_, and MSP3b (AUC value = 0.714–0.764) (**Figure 4A**, **Table 1**). The top 5 total IgG responses for classifying recent *P. vivax* infection were against the following: RBP2b_1986-2653_, MSP3a, MSP7B, and two Pv-fam-a proteins (PVX_096995, PVX_090265) (AUC value = 0.818–0.833) (**Figure 4B**, **Supplementary Table S5**). Individually, two Pv-fam-a proteins (PVX_125728, PVX_096995), RBP2b_1986-2653_, MSP3b, and MSP3a generated both IgG3 (AUC > 0.7) and total IgG (AUC > 0.8) that were good markers of recent *P. vivax* infection within the prior 9 months (**Supplementary Table S5**).

**Table 1:** AUC value of each *Plasmodium vivax* antigen-specific IgG3 response for classifying recent *P. vivax* infections occurring within a different range of time (1–9 months).

Antigens	AUC value								
	1 month	2 months	3 months	4 months	5 months	6 months	7 months	8 months	9 months
Pv-fam-a (PVX_125728)	0.661	0.702	0.703	0.714	0.723	0.729	0.723	0.753	0.764
Pv-fam-a (PVX_096995)	0.673	0.707	0.716	0.728	0.728	0.733	0.728	0.738	0.740
MSP5	0.659	0.683	0.683	0.701	0.706	0.707	0.706	0.717	0.724
RBP2b_1986-2653_	0.614	0.656	0.677	0.695	0.714	0.716	0.714	0.720	0.722
MSP3b	0.630	0.669	0.666	0.677	0.699	0.705	0.699	0.709	0.714
MSP3a	0.622	0.645	0.657	0.675	0.693	0.698	0.693	0.702	0.703
PVX_101530	0.605	0.646	0.646	0.648	0.666	0.677	0.666	0.681	0.686
Pv-fam-a	0.609	0.646	0.647	0.657	0.670	0.671	0.670	0.683	0.685
MSP1-19	0.628	0.649	0.666	0.667	0.669	0.674	0.669	0.671	0.676
RBP2b_161-1009_	0.620	0.659	0.657	0.667	0.677	0.678	0.677	0.677	0.675
MSP7L	0.603	0.645	0.641	0.650	0.657	0.664	0.657	0.667	0.673
MSP7B	0.614	0.623	0.626	0.637	0.656	0.664	0.656	0.664	0.672
CSP210	0.628	0.667	0.662	0.662	0.658	0.655	0.658	0.661	0.663
AMA1	0.598	0.634	0.644	0.642	0.650	0.653	0.650	0.651	0.655
Hypothetical protein	0.587	0.627	0.641	0.648	0.650	0.653	0.650	0.653	0.655
PVX_090970	0.587	0.619	0.625	0.629	0.643	0.651	0.643	0.652	0.654
RAMA	0.599	0.625	0.623	0.635	0.649	0.652	0.649	0.649	0.652
Pv-fam-a (PVX_092990)	0.594	0.634	0.635	0.645	0.645	0.648	0.645	0.650	0.648
MSP 8	0.583	0.611	0.619	0.636	0.637	0.645	0.637	0.644	0.647
PTEX150	0.619	0.642	0.644	0.652	0.651	0.651	0.651	0.641	0.646
s16	0.610	0.631	0.632	0.640	0.645	0.645	0.645	0.640	0.639
RON2	0.589	0.616	0.610	0.631	0.633	0.641	0.633	0.634	0.637
Exported protein	0.600	0.625	0.617	0.628	0.632	0.635	0.632	0.638	0.634
Pv-fam-a (PVX_088820)	0.571	0.605	0.604	0.615	0.622	0.620	0.622	0.628	0.625
Pv-fam-a (PVX_112670)	0.556	0.593	0.607	0.618	0.623	0.628	0.623	0.623	0.623
MSP 7	0.564	0.588	0.594	0.614	0.615	0.626	0.615	0.622	0.621
PVX_091710	0.564	0.606	0.602	0.605	0.616	0.624	0.616	0.616	0.617
SIAP2	0.570	0.590	0.593	0.593	0.604	0.605	0.604	0.600	0.602
MSP7F	0.555	0.556	0.563	0.559	0.567	0.565	0.567	0.571	0.571

The two color scheme highlights the lowest AUC values in red and the highest AUC values in blue.

Because IgG3 was short-lived, the classification performance of IgG3 was also assessed in a narrower time frame (from 1 to 8 months). However, the performance declined as the window of time to detect infection narrowed (**Table 1**). The same pattern was observed for total IgG (data not shown).

### Characteristics that affected the acquisition of IgG3 in the Peruvian cohort

Because of the heterogeneity in the IgG3 response in the Peruvian individuals, we explored the factors that affected the acquisition of IgG3 in this cohort as has previously been reported for total IgG (Rosado et al., 2021). Linear regression models were fitted using age, gender, community, and the number of blood-stage *P. vivax* infections in the past 13 months (**Table 2**). The IgG3 level increased with increasing age, being male, living in Lupuna, and having ≥3 blood-stage *P. vivax* infections. Age (in log_10_) was the only factor that was associated with the response of IgG3 to all 29 P*. vivax* antigens in the panel (coefficient range: 0.182–0.849, *p* < 0.01). Age also had the highest coefficients compared to other variables to most antigens. Being male was associated with higher IgG3 levels to nine *P. vivax* antigens (coefficient range: 0.084–0.193, *p* < 0.05). Living in Lupuna was associated with higher IgG3 to 27 out of 29 antigens (coefficient range: 0.121–0.688, *p* < 0.01). Having ≥3 blood-stage infections within 13 months was positively correlated to higher IgG3 level to 15 P*. vivax* antigens (coefficient range: 0.125–0.347, *p* < 0.05).

**Table 2:** Multivariable linear regression model of the effect of epidemiological factors on IgG3 antibody level in the Peruvian cohort.

Antigen	Age, Log_10_	Gender(Ref = female)	Community(Ref = Cahuide)	> 3 infection^¶^(Ref = No)
		Coefficient (95% CI)	Coefficient (95% CI)	Coefficient (95% CI)	Coefficient (95% CI)	
AMA 1	0.556***(0.383, 0.730)		0.354***(0.225, 0.482)	
CSP210	0.347***(0.209, 0.486)		0.254***(0.162, 0.346)	0.126*(0.005, 0.248)
exported	0.341***(0.201, 0.481)		0.176***(0.081, 0,271)	
hypothetical protein	0.462***(0.297, 0.626)		0.229***(0.107, 0,350)	0.147*(0.002, 0.292)
MSP 5	0.743***(0.546, 0.940)		0.491***(0.339, 0.643)	0.266**(0.073, 0.459)
MSP 7	0.368***(0.197, 0.539)	0.153*(0.023, 0.283)	0.178*(0.042, 0.315)	0.265**(0.087, 0.443)
MSP1-19	0.366***(0.176, 0.555)		0.249**(0.107, 0.390)	
MSP3a	0.497***(0.313, 0.682)	0.160*(0.0306, 0.289)	0.409***(0.270, 0.548)	0.272**(0.094, 0.449)
MSP3b	0.550***(0.365, 0.734)		0.330***(0.191, 0.469)	0.347***(0.171, 0.523)
MSP7B	0.849***(0.622, 1.076)	0.193*(0.028, 0.357)	0.688***(0.516, 0.861)	0.293**(0.072, 0.514)
MSP7F	0.184***(0.087, 0.281)		0.121***(0.058, 0.184)	
MSP7L	0.182**(0.078, 0.286)			
MSP8	0.337***(0.183, 0.490)	0.113*(0.002, 0.225)	0.323***(0.216, 0.431)	0.256**(0.103, 0.410)
PTEX150	0.385***(0.242, 0.529)		0.207***(0.102, 0.312)	0.154*(0.025, 0.283)
Pv-fam-a (PVX_088820)	0.311***(0.198, 0.425)		0.160***(0.079, 0.241)	0.125*(0.024, 0.226)
Pv-fam-a (PVX_090265)	0.295***(0134, 0.456)		0.203**(0.088, 0.3170	0.206**(0.072, 0.340)
Pv-fam-a (PVX_092990)	0.202**(0.064, 0.341)	0.169***(0.075, 0.264)	0.207***(0.110, 0.304)	
Pv-fam-a (PVX_096995)	0.536***(0.347, 0.725)		0.318***(0.177, 0.459)	0.333***(0.155, 0.510)
Pv-fam-a (PVX_112670)	0.193**(0.077, 0.309)			
Pv-fam-a (PVX_125728)	0.249***(0.139, 0.360)	0.084*(0.006, 0.161)	0.183***(0.104, 0.261)	
PVX_090970	0.368***(0.234, 0.502)	0.113*(0.025, 0.201)	0.173***(0.081, 0.265)	
PVX_091710	0.233***(0.112, 0.354)	0.150**(0.065, 0.234)	0.126**(0.044, 0.207)	
PVX_101530	0.281***(0.145, 0.417)		0.226***(0.130, 0.321)	
RAMA	0.196***(0.092, 0.301)		0.142***(0.072, 0.213)	
RBP2b_161-1009_	0.312***(0.167, 0.458)	0.102*(0.000, 0.204)	0.190***(0.085, 0.296)	0.185**(0.055, 0.314)
RBP2b_1986-2653_	0.285***(0.142, 0.428)		0.301***(0.199, 0.404)	0.173**(0.048, 0.298)
RON2	0.257***(0.118, 0.396)		0.292***(0.195, 0.388)	
s16	0.271**(0.111, 0.430)		0.286***(0.171, 0.401)	
SIAP2	0.243**(0.100, 0,386)		0.213***(0.110, 0.315)	0.146*(0.026, 0.265)

95% CI, 95% confidence interval.

*p < 0.05; **p < 0.01; ***p < 0.001.

a Plasmodium vivax-positive qPCR result.

Only significant associations are shown.

The proportion of individuals with *P. vivax* infections was higher in Lupuna (84.05%) than in Cahuide (66.09%) as detected by qPCR in the last 13 months (Rosado et al., 2021). Hence, we assessed the classification performance of IgG3 and total IgG using the subset of individuals living in Lupuna (**Supplementary Table S5**). Classification performance using IgG3 on the individuals in Lupuna was higher than when using individuals from the whole cohort (AUC range 0.589–0.864 and 0.571–0.764, respectively). However, the classification performance of total IgG also improved (AUC range 0.83–0.912 Lupuna vs. 0.584–0.838 whole cohort), providing evidence that overall total IgG is a better marker of recent exposure than IgG3 in regions with moderate transmission intensity.

### Comparison of total IgG data acquired using non-magnetic and magnetic beads

**Figure 5:**
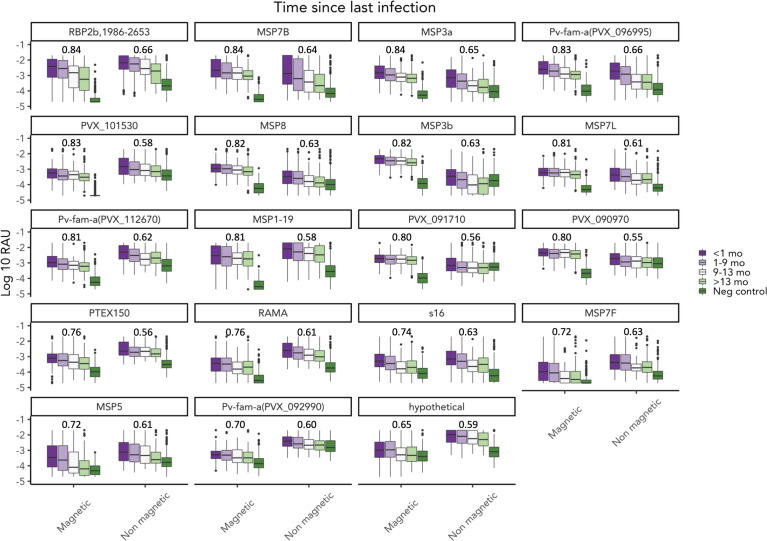
Comparison of total IgG measured using magnetic beads versus non-magnetic beads. Total IgG to 19 *P. vivax* antigens was measured using magnetic and non-magnetic beads in the multiplex assay. Total IgG was measured in the Peruvian cohort (n = 590) and negative controls (n = 274). AUC value was noted in the figure. Non-magnetic bead AUC value was obtained from a published paper by Rosado et al.

The strong performance of total IgG in this Peruvian cohort for classifying recent *P. vivax* infections in the prior 9 months was unexpected. A previous work had indicated total IgG was a poor performer in this cohort of moderate transmission intensity, using a number of the same *P. vivax* antigens and the same methods for testing the accuracy of classification (Rosado et al., 2021). A key difference between the two studies was the use of magnetic beads in the current work compared to non-magnetic beads in the prior work. The antibody levels generated in the Peruvian individuals, to the same *P. vivax* antigens, were therefore compared between the current data (magnetic beads) and the prior dataset (non-magnetic beads (Rosado et al., 2021)). In the Peruvian individuals, there were moderate to strong correlations in total IgG antibody levels across the 19 P*. vivax* antigens in common (*r* values 0.38–0.91) (**Supplementary Figure S6**). However, within the negative controls, the correlations were weaker (0.16–0.62), with no correlation for one antigen (PVX_101530, *r* = −0.015, *p* = 0.81) (**Supplementary Figure S7**). There were lower antibody levels detected in the malaria-naive controls for most of the *P. vivax* antigens assessed when using magnetic beads (**Figure 5**), which has resulted in a better signal-to-background ratio (when comparing antibody levels in those with recent infections to the negative controls), which in turn has likely contributed to the better classification performance of total IgG in the current study. This is further demonstrated through a focused analysis on the top performing antigen using total IgG, RBP2b_1986-2653_. The ROC curves for RBP2b_1986-2653_ generated using non-magnetic beads and magnetic beads are compared in **Supplementary Figure S8**, with the breakdown of the classification demonstrating that the improved overall AUC value when using magnetic beads (0.84 vs. 0.66) is due to improved performance in classifying the negative controls as not recently exposed [88.7% (243/274) correctly classified using non-magnetic bead data vs. 98.6% (270/274) with magnetic bead data]. There was no improvement evident in the classification of the Peruvian individuals with 66.8% (394/590) correctly classified when using the non-magnetic bead data and 66.6% (393/590) when using magnetic bead data (**Supplementary Figures S8B, C**).

## Discussion

In countries endemic to both *P. vivax* and *P. falciparum*, *P. vivax* is proving to be a significant challenge for control and elimination. New strategies for rapidly reducing the transmission of *P. vivax* need to be considered, such as serological testing and treatment (seroTAT) (Tayipto et al., 2022). The current development of seroTAT has focused on applying this strategy in low transmission settings. In settings with higher transmission, there is concern that serological exposure markers will be less accurate (Rosado et al., 2021) given that individuals will likely have had higher levels of past exposure to *P. vivax* and therefore longer-lived IgG responses (Longley et al., 2017; Rosado et al., 2021). In the current study, the goal was to first characterize the longevity of total IgG, IgG1, IgG3, and IgM following asymptomatic *P. vivax* infections in a setting of moderate–high transmission intensity and then to assess the classification performance of the antibody with the most suitable longevity profile (i.e., short-lived). A limitation in the approach for our study was the need to characterize longevity and then assess classification performance using cohorts from two discrete populations (for both age and geography) (Rosado et al., 2021, Robinson et al., 2015). While both settings and cohorts had higher transmission than our prior work in Thailand, Brazil, and the Solomon Islands (Longley et al., 2020), there were also differences between them with the PNG community having even higher transmission at the time of the study than the Peruvian communities (based on *P. vivax* prevalence by PCR) (Rosado et al., 2021, Robinson et al., 2015).

As expected, total IgG responses in PNG child1ren to most of the *P. vivax* antigens in the panel were maintained above baseline over 36 weeks. However, the response was only slightly above the seropositivity cutoff, except IgG to RBP2b_1986-2653_, which was highly immunogenic. This observation is consistent with prior studies highlighting RBP2b as highly immunogenic (Liu et al., 2022) and indeed the top marker of recent exposure when using total IgG in low transmission settings (Longley et al., 2020). IgM responses in PNG children were of relatively low magnitude compared to the negative control panels, with seropositivity rates at the time of infection <50% for all but two antigens, but the levels were consistently maintained over the 36 weeks. We have previously observed long-lived IgM responses following clinical *P. vivax* infections in a low transmission setting (Liu et al., 2022), and data on immune responses to *P. falciparum* indicate that IgM is a prominent feature of the antibody response to malaria, even in those with repeated infections over time (Boyle et al., 2019). IgG1 was more immunogenic and had higher seropositivity than IgG3 in the PNG children cohort, which could be explained by these children not yet undergoing the IgG subclass switch from IgG1 to IgG3 in relation to age (Fernandez-Becerra et al., 2010; França et al., 2016). IgG1 responses were maintained and slowly declined over time, mirroring total IgG, while the IgG3 responses that were elicited declined to background 6–8 weeks after enrolment. Among the tested antibodies, IgG3 had the most appropriate longevity profile for further testing as a marker of recent *P. vivax* exposure in areas with moderate/moderate–high transmission intensity.

To test the ability of antigen-specific IgG3 antibody responses to classify individuals as recently infected with *P. vivax* in the prior 9 months, samples from a Peruvian cohort were used. The ability of total IgG to classify recent infections in this cohort has previously been tested and shown to be suboptimal compared to classification in low transmission settings (Rosado et al., 2021). Overall, IgG3 was not strongly induced in the Peruvian cohort and, thus, not surprisingly, was a poor classifier of recent exposure. The performance of total IgG to most *P. vivax* antigens was more accurate than IgG3. The top 5 IgG3 markers were able to classify infection within 9 months with AUC values of >0.7, while the top 5 total IgG markers had AUC values of >0.8. Among the top 5 performers, IgG3 to MSP5 and one Pv-fam-a protein (PVX_125728) also had the highest IgG3 seropositivity rates of 41.2% and 62%, respectively, in the Peruvian cohort. Other top 5 performers, IgG3 to another Pv-fam-a protein (PVX_096995) and MSP3b, had the highest mean level of IgG3 detected across all Peruvian samples. On the contrary, despite being one of the best markers of recent *P. vivax* infection using IgG3, the seropositivity rates of IgG3 to RBP2b_1986-2653_ were the lowest in the Peruvian cohort, either in those infected in the prior 9 months or among all participants in the cohort. IgG3 antibodies against RBP2b_1986-2653_ were clearly induced; however, the seropositivity cutoff set was high given a large spread of IgG3 levels against this protein in the negative control panels. This is thus a factor that could be further optimized through construct design and/or purification. Overall, the top IgG3 performers for classifying recent *P. vivax* infections in the Peruvian cohort had characteristics of high seropositivity or high mean magnitude, except for RBP2b_1986-2653_. Despite IgG3 levels being short-lived in PNG children, IgG3 classification of recent prior *P. vivax* infections in Peru was poorer for shorter intervals of classification (i.e., 1–8 vs. 9 months). The reason for this finding is currently unclear but could be influenced by different patterns of antibody kinetics in PNG children versus the Peruvians, despite similar levels of transmission (moderate/moderate–high). This is a challenge and is also highlighted by differences in the results for total IgG between the PNG and Peruvian cohorts and suggests that perhaps antibody kinetics need to be assessed in the same target population as where the serological exposure markers would be implemented; however, this is not always possible depending on the design of studies that have been conducted.

To further understand the acquisition of IgG3, epidemiologic variables that could influence the results were assessed. IgG1 switching to IgG3 has been associated with age as reported in other studies (Fernandez-Becerra et al., 2010; França et al., 2016). Age had the strongest association with IgG3 levels (coefficient range: 0.182–0.849, *p* < 0.01) compared to gender, location, and number of previous *P. vivax* infections in the Peruvian cohort. People living in Lupuna also had significantly higher IgG3 levels to 28 out of 29 P*. vivax* proteins. In the last 13 months, people in Lupuna had 18% more *P. vivax* infections than those in Cahuide (Rosado et al., 2021). Individuals living in Lupuna also had more past exposure at enrolment (Rosas-Aguirre et al., 2021). This correlates with the result in this study that showed that individuals who had at least three blood-stage *P. vivax* infections had high levels of IgG3. A prior study in the same Peruvian cohort also found that total IgG increased with increasing age and number of blood-stage *P. vivax* infections (Rosado et al., 2021). Gender, on the other hand, was associated weakly with the level of IgG3. Together, the data suggest that a high level of prior exposure to *P. vivax* is required to gain IgG3 antibodies, with too few individuals in the Peruvian cohort acquiring enough IgG3 to enable accurate classification of recent *P. vivax* exposure with this biomarker.

Total IgG to the *P. vivax* antigen panel in the Peruvian cohort resulted in better classification performance of recent *P. vivax* infections, with AUC >0.8, than that obtained in the previous study of the same cohort (<0.7) (Rosado et al., 2021). There were 19 antigens in common between the two panels and similar top performers: total IgG to RBP2b_1986-2653_, MSP3a, MSP7B, and one Pv-fam-a protein (PVX_096995). Importantly, the *P. vivax* antigens in common between the two studies were expressed using the same constructs and expression systems. The key difference was the use of a magnetic bead-based assay in the current study compared to a non-magnetic bead-based assay in the earlier study. Systematic comparisons of the use of non-magnetic versus magnetic beads for the multiplexed assays have demonstrated that moderate–strong correlations in the data can be expected (Ondigo et al., 2019; Mazhari et al., 2020), dependent on the antigen. The current results are in support of this finding. However, the correlation was weaker in the malaria-naive negative controls. The magnetic bead assay had a lower background, suggesting that a better signal-to-background ratio increases the classification accuracy [a trend we have observed previously (Longley et al., 2020)]. The non-magnetic bead assay was run on a BioPlex-200 instrument with the high RP1 (PMT) option selected, which is recommended when running plasma or sera samples. This option amplifies the signal resulting in improved sensitivity and, thus, is a likely contributing factor to the higher antibody levels detected in the negative controls using this system. Alternatively, the improved methods for plate washing that are enabled through the use of magnetic beads (such as an automatic plate washer) may also contribute to the lower background in the magnetic bead assay. Ultimately, the improved AUC was due to better classification of the negative control samples as not recently exposed to *P. vivax* and not due to improvements in classifying the Peruvian individuals as recently or not recently exposed. This highlights the importance of assessing classification algorithm performance specifically within defined groups (such as in **Figure S8**) and leads to the same finding as in our original study: the performance of *P. vivax* serological exposure markers (using total IgG) is poorer in moderate transmission settings than low transmission settings. The current study additionally shows that IgG3 is also inadequate for classifying recent exposure with high accuracy.

In exploring the use of IgG3 as a biomarker of recent *P. vivax* infection, further work is needed to assess the combinations of IgG3 responses to multiple *P. vivax* antigens. Prior evidence has shown that using combined total IgG antibody responses to more than five *P. vivax* antigens was a better marker of recent infection than to one alone (Longley et al., 2020), so a combination of antigen-specific antibodies may increase the performance of IgG3. Rescreening a larger set of *P. vivax* proteins may also guide finding a better antigen that generates a stronger IgG3 response in individuals living in endemic areas with moderate transmission intensity. However, the heterogeneity seen for the IgG3 antibody profile, which was largely dependent on age and the level of previous exposure, demonstrates that IgG3 on its own is not well suited as a recent exposure marker in this epidemiological setting of Peru. Furthermore, IgG3 antibody kinetics in PNG children may differ to those in Peruvian individuals of all ages, which suggests that either i) other antibody biomarkers should be directly screened in the Peruvian cohort or ii) antibody kinetics need to be defined in Peruvian studies. Ultimately, the *P. vivax* serological exposure markers are designed for use in low transmission settings [where they perform very well (Longley et al., 2020)], and further consideration and optimization, and comparison to available alternatives, would be required before supporting their use in higher transmission settings.

## Data availability statement

The original contributions presented in the study are included in the article/**Supplementary Material**. Further inquiries can be directed to the corresponding author.

## Ethics statement

The studies involving human participants were reviewed and approved by Walter and Eliza Hall Institute Human Research Ethics Committee, PNG Institute of Medical Research Institutional Review Board, PNG Medical Advisory Committee, Ethics Committee of Basel, Ethics Review Board of Universidad Peruana Cayetano Heredia, University of California San Diego Human Subjects Protection Program. Written informed consent to participate in this study was provided by the participants’ legal guardian/next of kin.

## Author contributions

YT and RL wrote the first draft of the manuscript. YT generated data from the samples and conducted data analysis with the support of JR, RL, MW, and IM. JR, DG, BK, and LR collected the data and samples in the field. JH, DO, JB, ET, TT, and MH expressed proteins. All authors have contributed to the final version of the manuscript.

## Funding

YT received an Australia Award from the Australian Department of Foreign Affairs and Trade to support her Master of Biomedical Science research. This work was supported by an Australian National Health and Medical Research Council (NHMRC) Investigator Grant (#1173210 to RL and #1173046 to JB). The PNG longitudinal cohort study was funded by NIH U19 AI089686. The Peruvian samples were collected under the ICEMR program (U19AI089681). LR was supported by NHMRC grants #1161627 and #1016443. IM was also supported by the NHRMC (grants #1092789, #1134989, #1132975, and #1043345). We acknowledge the support of the Victorian State Government Operational Infrastructure Support and Australian Government NHMRC IRIISS.

## Acknowledgments

The following people provided drafts of R scripts, PRISM file, or STATA do-file for data analysis: Dr. Connie Li Wai Suen (five-parameter logistic regression model) and Dr. Eamon Conway (antibody kinetics graphs). We thank Jessica Brewster for the assistance with coupling some beads for the multiplex assay. We thank all the teams in PNG and Peru involved in collecting samples or data from the cohort studies.

## Conflict of interest

RL, MW, TT, and IM are inventors on patent PCT/US17/67926 on a system, method, apparatus, and diagnostic test for *P. vivax*. Author MH was employed by CellFree Sciences Co., Ltd., Yokohama, Japan.

The remaining authors declare that the research was conducted in the absence of any commercial or financial relationships that could be construed as a potential conflict of interest.

## Publisher’s note

All claims expressed in this article are solely those of the authors and do not necessarily represent those of their affiliated organizations, or those of the publisher, the editors and the reviewers. Any product that may be evaluated in this article, or claim that may be made by its manufacturer, is not guaranteed or endorsed by the publisher.
