# Supplementary material for: Assessment of IgG3 as a serological exposure marker for Plasmodium vivax in areas with moderate–high malaria transmission intensity
Source: Front Cell Infect Microbiol. 2022 Aug 9;12:950909. doi: 10.3389/fcimb.2022.950909 (PMC9395743; doi:10.3389/fcimb.2022.950909)
Supplement: Supplementary file 1 [file Presentation_1.pdf]

Supplementary Material

1 Supplementary Data

Supplementary Data Files are attached separately.

Supplementary Data 1: All RAU data from the PNG child cohort and the negative control panels. “Type_antibody” specifies the biomarker (i.e. total IgG, IgG1 etc).

Supplementary Data 2: All RAU data from the Peruvian cohort (log10 transformed). Sheet 1 contains IgG data and sheet 2 contains IgG3 data. Sheet 3 includes the data dictionary.

2 Supplementary Figures and Tables

2.1 Supplementary Figures

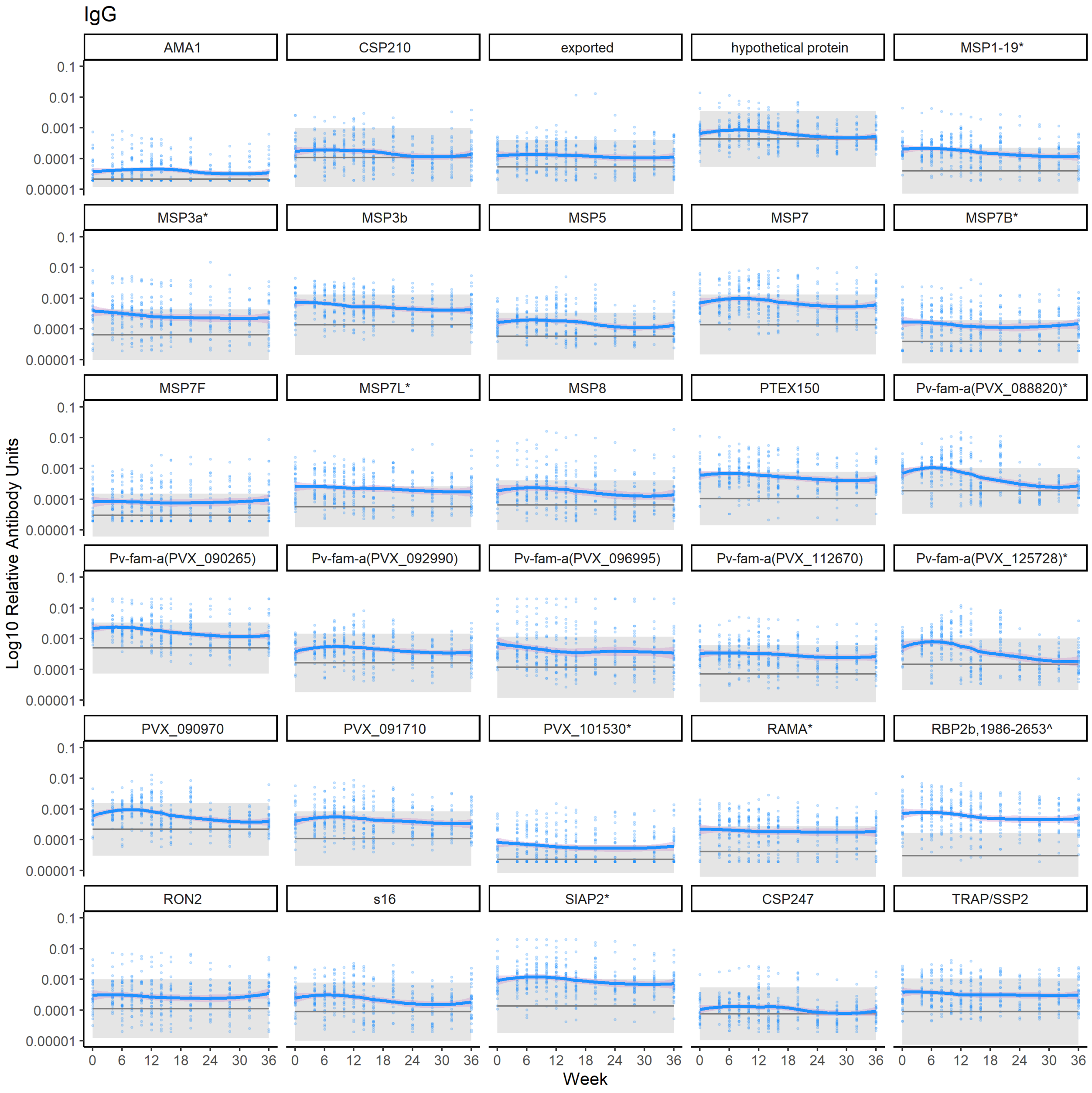

Supplementary Figure S1. The kinetics of total IgG against 30 *P. vivax* antigens following asymptomatic *P. vivax* infections in PNG children.

Total IgG antibody responses against 30 *P. vivax* antigens following asymptomatic *P. vivax* infections in PNG children (n=31) over 36 weeks were measured using a multiplex assay. Data is presented in RAU (in log 10). Each dot point shows the individual antibody response to each *P. vivax* antigen measured at each timepoint. Locally estimated scatterplot smoothing (LOESS) lines and 95% confidence intervals were plotted over time. Negative control (grey line) was the average antibody response of naïve individuals (n=274) and the grey ribbon was set as 2 SD from the average negative control. Anti-antigen antibody with LOESS-smoothed line that was seropositive at week 0 and became seronegative within 9 months are indicated by an asterix (*). Anti-antigen antibody with LOESS-smoothed line that was seropositive for at least 36 weeks is indicated by an up arrow (^).

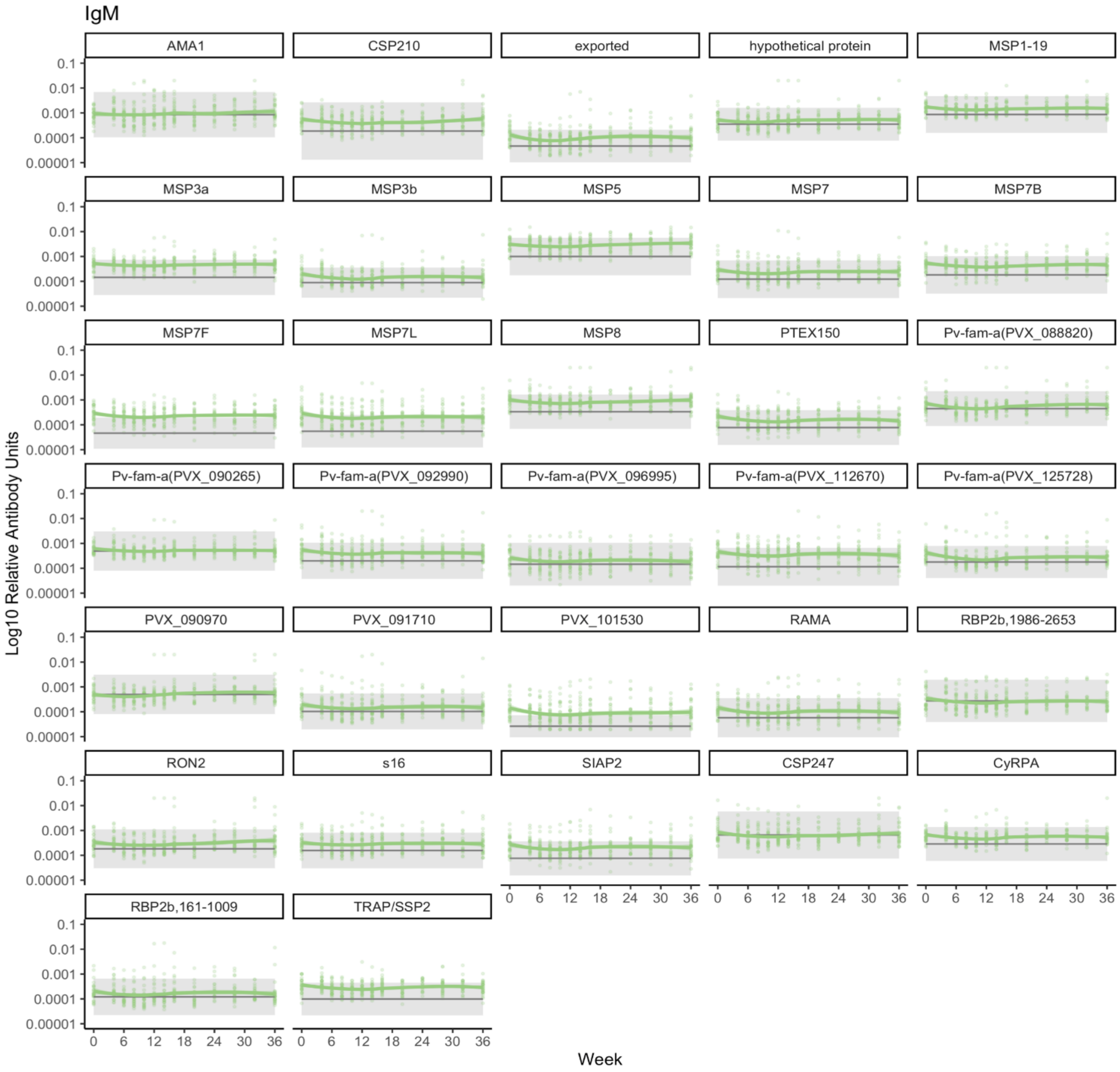

Supplementary Figure S2. The kinetics of IgM against 32 *P. vivax* antigens following asymptomatic *P. vivax* infections in PNG children.

IgM antibody responses against 32 *P. vivax* antigens following asymptomatic *P. vivax* infections in PNG children (n=31) over 36 weeks, measured using a multiplex assay. Data are presented in RAU (in log 10). Each dot point shows the individual IgM antibody response to *P. vivax* antigens measured at each timepoint. LOESS-smoothed line and 95% confidence intervals were plotted over time. Negative control (grey line) was the average antibody response of naïve individuals (n=260) and the grey ribbon shows 2 SD from average negative control. No anti-antigen antibody with LOESS-smoothed line that was seropositive at week 0 and became seronegative within 9 months.

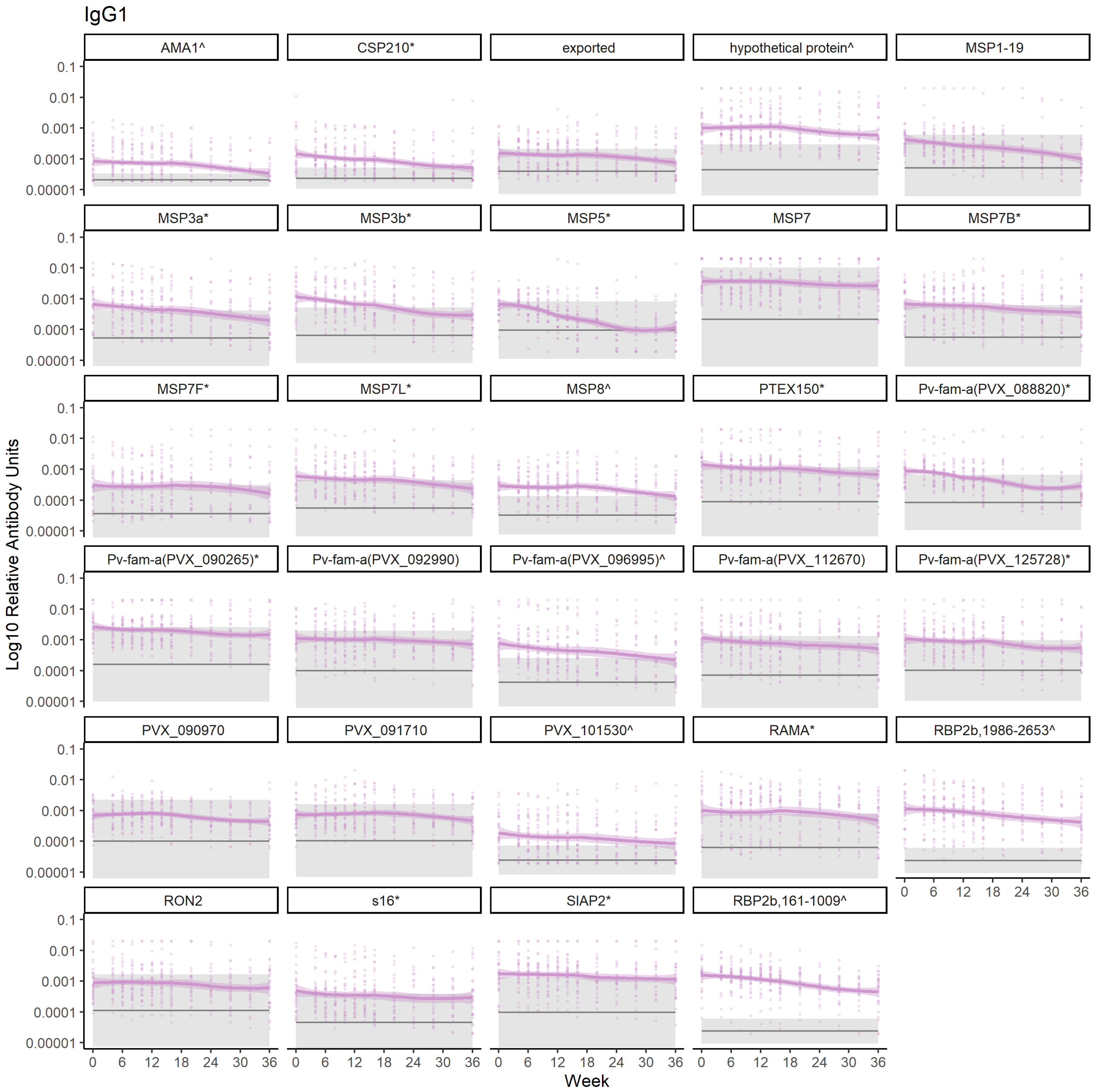

Supplementary Figure S3. The kinetics of IgG1 against 29 *P. vivax* antigens following asymptomatic *P. vivax* infections in PNG.

IgG1 antibody response against 29 *P. vivax* antigens following asymptomatic *P. vivax* infections in PNG children (n=31) over 36 weeks, measured using a multiplex assay. Data are presented in RAU (in log 10). Each dot point shows the individual antibody response to *P. vivax* antigens measured at each timepoint. LOESS-smoothed lines and 95% confidence intervals were plotted over time. Negative control (grey line) was the average antibody response of naïve individuals (n=248) and the grey ribbon shows 2 SD from average negative control. Anti-antigen antibody with LOESS-smoothed line that was seropositive at week 0 and became seronegative within 9 months are indicated by an asterix (*). Anti-antigen antibody with LOESS-smoothed line that was seropositive for at least 36 weeks is indicated by an up arrow (^).

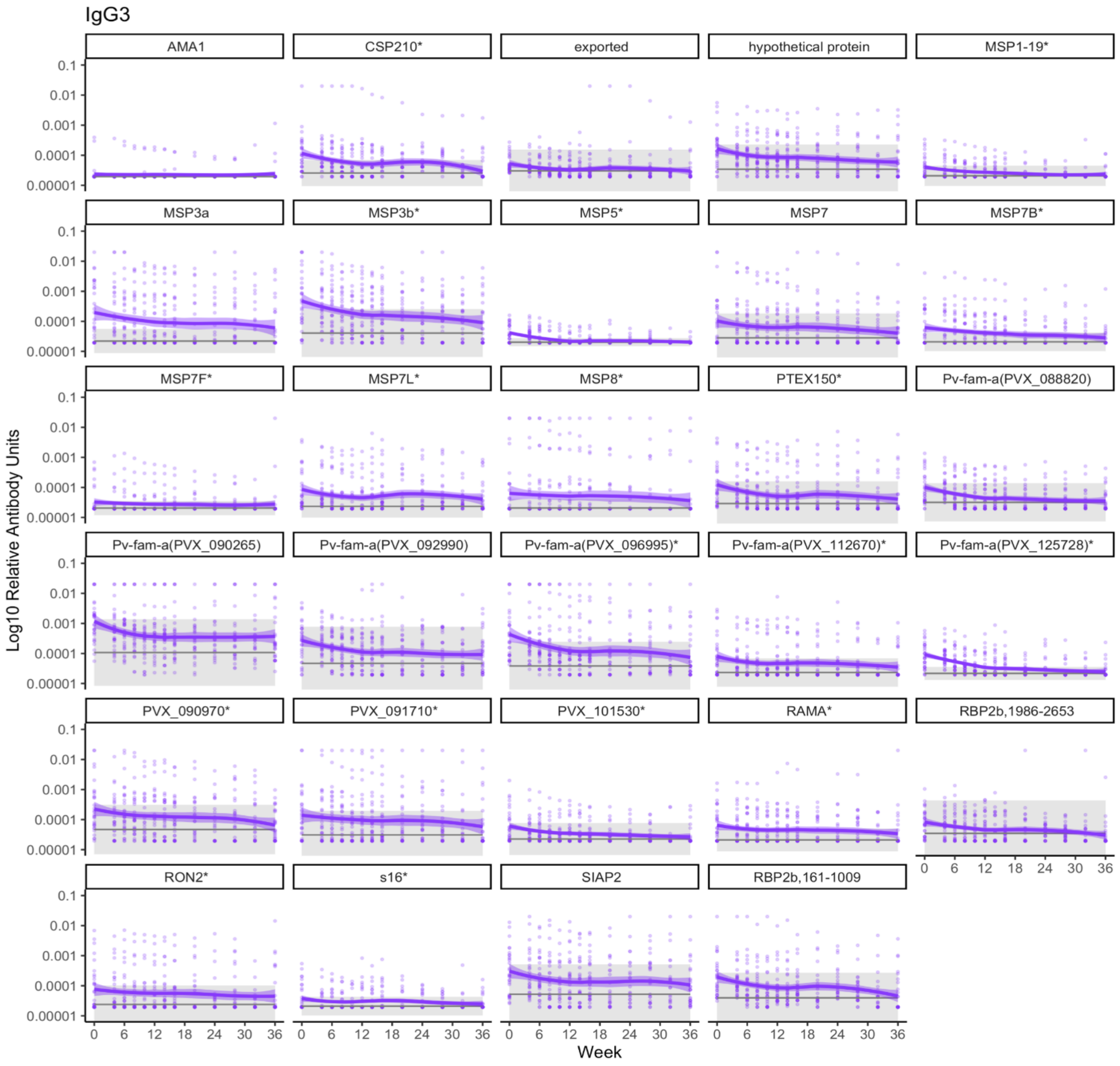

Supplementary Figure S4. The kinetics of IgG3 against 29 *P. vivax* antigens following asymptomatic *P. vivax* infections in PNG.

IgG3 antibody response against 29 *P. vivax* antigens following asymptomatic *P. vivax* infections in PNG children (n=31) over 36 weeks, measured using a multiplex assay. Data are presented in RAU (in log 10). Each dot point shows the individual antibody response to *P. vivax* antigens measured at each timepoint. LOESS-smoothed lines and 95% confidence intervals were plotted over time. Negative control (grey line) was the average antibody response of naïve individuals (n=256) and the grey ribbon shows 2 SD from average negative control. Anti-antigen antibody with LOESS-smoothed line that was seropositive at week 0 and became seronegative within 9 months are indicated by an asterix (*).

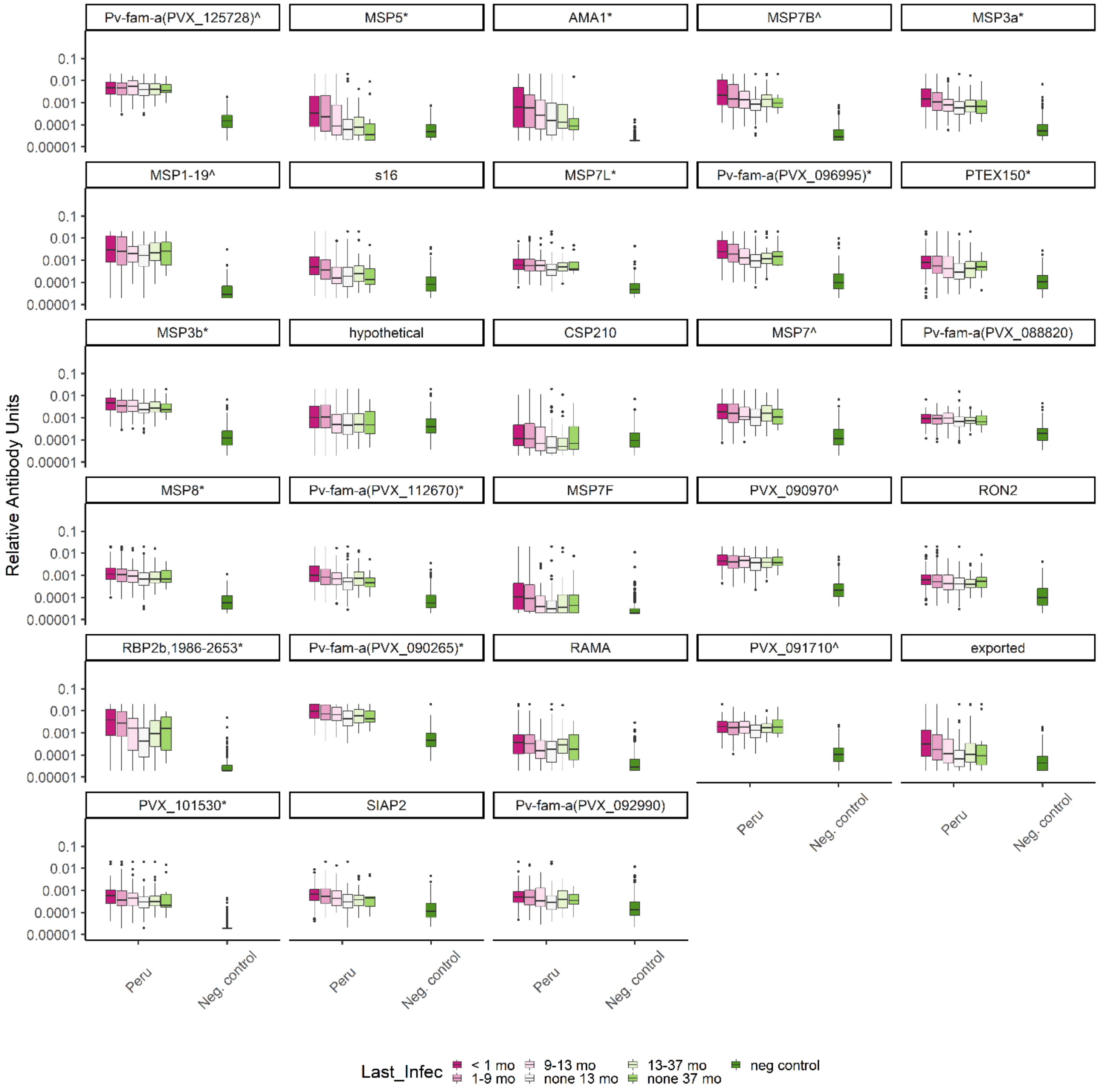

Supplementary Figure S5. Total IgG responses to 28 *P. vivax* antigens in Peruvian cohort based on time since last infected with *P. vivax.*

Total IgG responses to 28 *P. vivax* antigens were measured at the end of study of a Peruvian cohort (n=590)). Antibody responses were presented in relative antibody unit (log 10) in median with interquartile range. Individuals were grouped based on the last *P. vivax* infection status as measured by qPCR. Antigen-specific IgG were ordered based on decreasing seropositivity to IgG3 to reflect Fig 3. Anti-antigen antibody that was significantly higher in individuals with infections in the prior 9 months compared to individuals with no infections or infections >9 months ago are indicated by an

asterix (*) (student's t-test, $p<0.05$). Anti-antigen antibody with consistent IgG levels above background, regardless of recency of infection are indicated by ^.

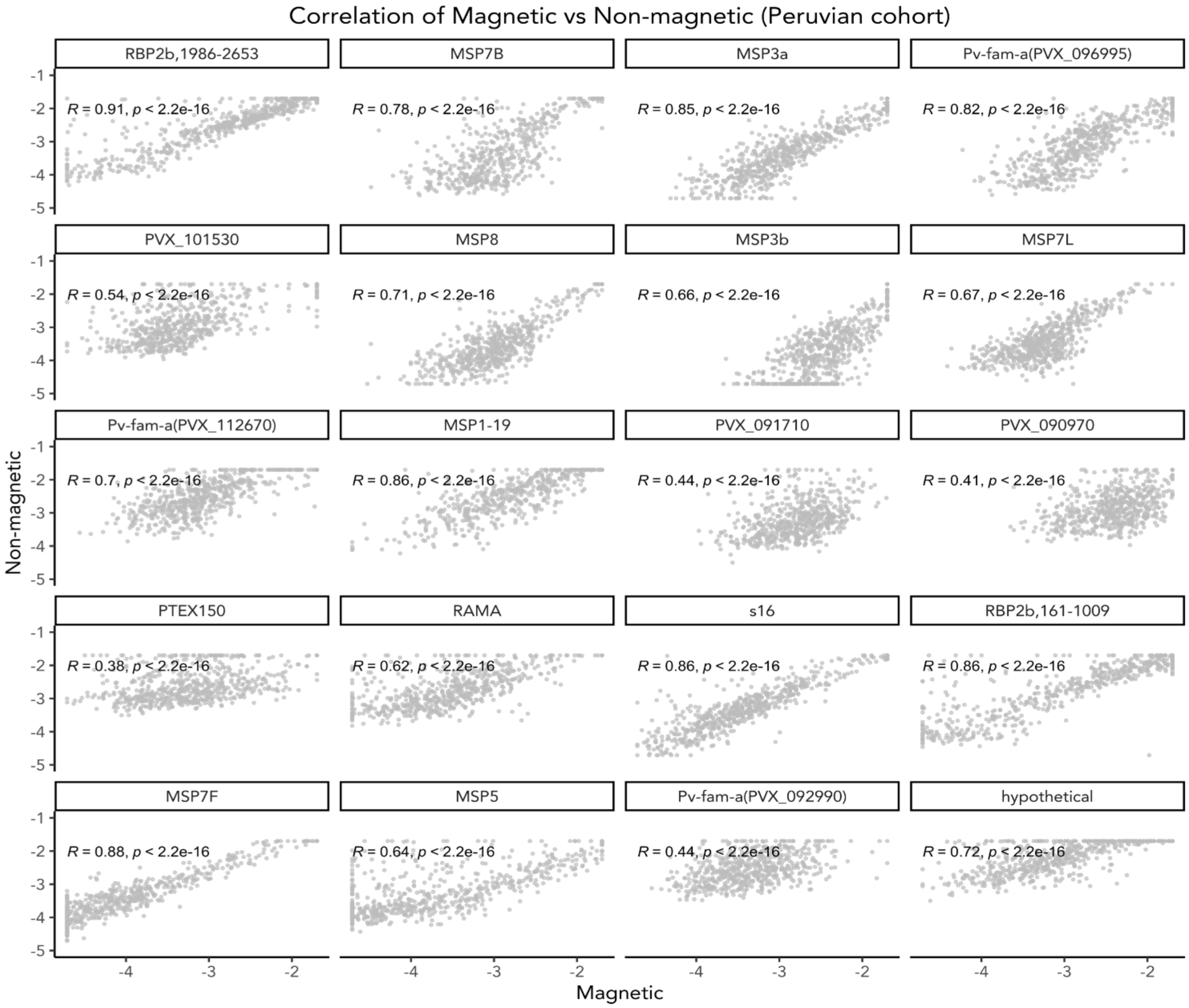

Supplementary Figure S6. Correlation of total IgG antibodies to 20 *P. vivax* antigens measured in Peruvian cohort using magnetic and non-magnetic bead in multiplex assay

Total IgG antibodies to 20 *P. vivax* antigens were measured in Peruvian cohort using magnetic and non-magnetic beads in the Peruvian cohort (n=590). Antibodies level is represented in relative antibody unit. Correlation is analysed using a Spearman’s rank correlation.

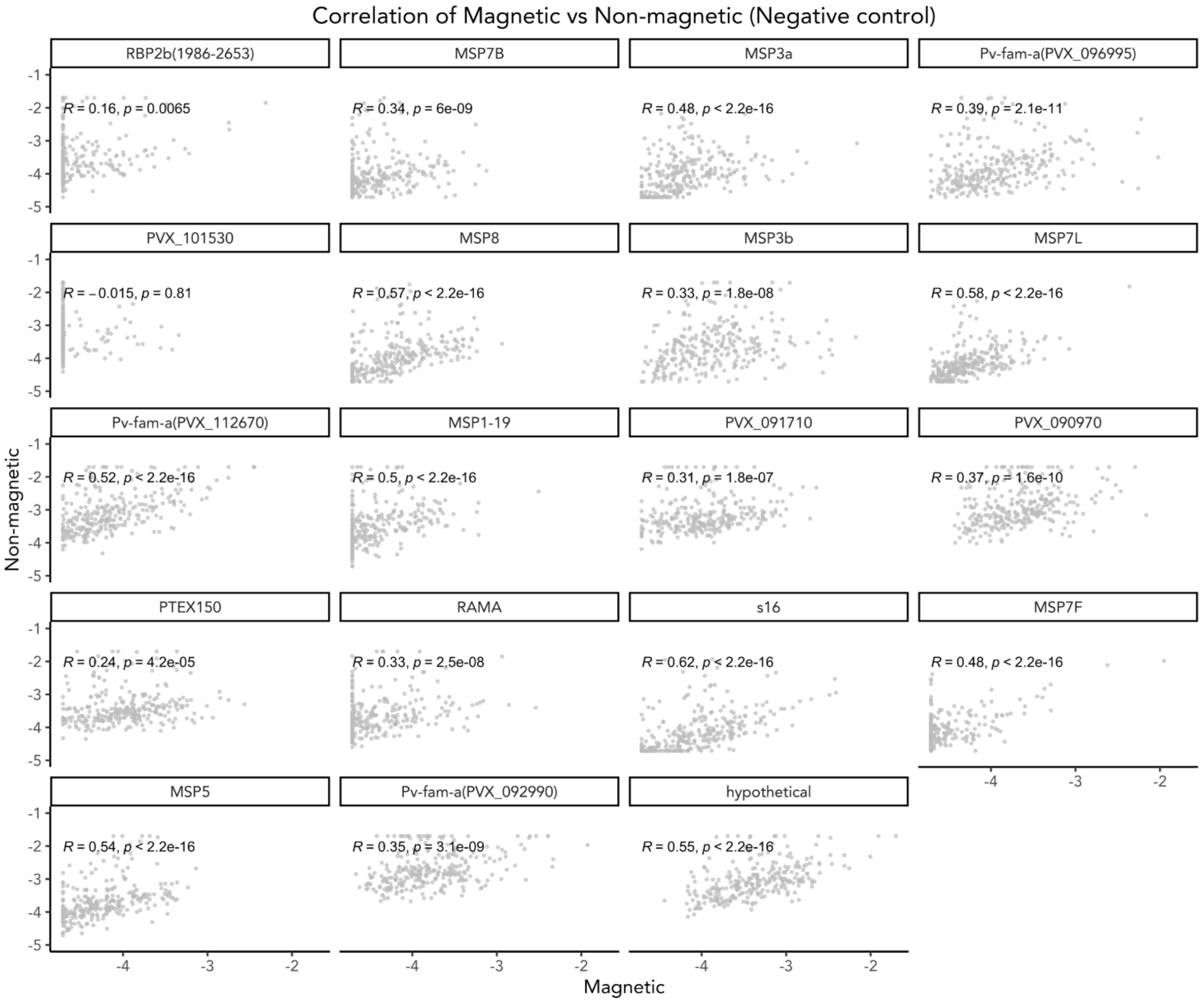

Supplementary Figure S7. Correlation of total IgG antibodies to 19 *P. vivax* antigens measured in negative controls using magnetic and non-magnetic bead in multiplex assay

Total IgG antibodies to 19 *P. vivax* antigens were measured in negative controls using magnetic and non-magnetic beads in the Peruvian cohort (n=590). Antibodies level is represented in relative antibody units. Correlations were analysed using a Spearman's rank correlation.

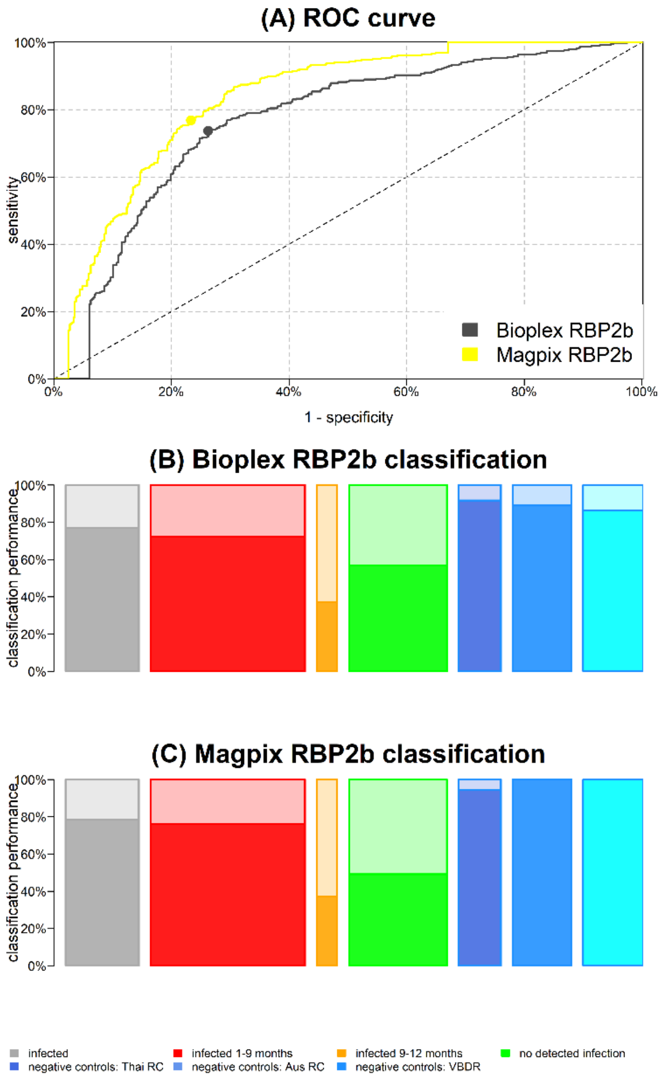

Supplementary Figure S8. Comparison of RBP2b$_{1986\text{-}2653}$ classification performance for identifying individuals with *P. vivax* infections in the last 9 months generated using non-magnetic beads and magnetic beads in Peruvian individuals.

(a) ROC curves for RBP2b$_{1986\text{-}2653}$ generated using non-magnetic beads and magnetic beads; (b) Bioplex RBP2b$_{1986\text{-}2653}$ classification performance; (c) Magpix RBP2b$_{1986\text{-}2653}$ classification performance. The size of the bar corresponds to the number of samples in each category. The darker colour represents the proportion of correctly classified individuals, and the lighter colour represents the proportion of mis-classified individuals.

2.2 Supplementary Tables

Supplementary Table S1. List of *P. vivax* antigens used for detecting antibodies in multiplex assay. Protein amount signifies the amount of protein added to a bulk coupling of magnetic beads as described in the methods. In the current study double the optimisied amount of protein was added compared to usual (see Mazhari et al PLOS One 2020 in main reference list), as a modification required to measure the presence of functional antibody responses. Log-linear standard curves were still achieved for all proteins. Abbreviations: aa=amino acid; WGCF= wheat germ cell-free system. Annotations: [a]=PlasmoDB.

Protein	PlasmoDB ID[a]	Construct aa (size)	Protein amount (ug)	Expression System
sexual stage antigen s16	PVX_000930	31-end (110)	4	WGCF expression
exported protein	PVX_003555	434-1075 (642)	2	WGCF expression
merozoite surface protein (MSP) 5	PVX_003770	23-365 (343)	0.08	WGCF expression
MSP7	PVX_082645	23-end (355)	32	WGCF expression
MSP7B	PVX_082650	24-end (429)	16	WGCF expression
MSP7F	PVX_082670	24-end (388)	2	WGCF expression
MSP7L	PVX_082700	23-end (397)	3	WGCF expression
thrombospondin-related anonymous protein (TRAP/SSP2)	PVX_082735	26-493 (468)	4	WGCF expression
PTEX150	PVX_084720	24-908 (885)	4	WGCF expression
rhoptry-associated membrane antigen (RAMA)	PVX_087885	462-730 (269)	2.4	WGCF expression
Pv-fam-a	PVX_088820	58-end (259)	64	WGCF expression
associated protein 2 (SIAP2)	PVX_088860	33-end (380)	16	WGCF expression
cysteine-rich protective antigen (CyRPA)	PVX_090240	27-366 (340)	4	Baculovirus
Pv-fam-a	PVX_090265	1-326 (326)	32	WGCF expression
hypothetical protein	PVX_090970	20-254 (235)	24	WGCF expression
hypothetical protein	PVX_091710	26-884 (859)	4	WGCF expression
apical membrane antigen 1 (AMA1)	PVX_092275	1-463 (464)	4	Expi293
Pv-fam-a	PVX_092990	1126-1414 (289)	32	WGCF expression
reticulocyte binding protein 2b (RBP2b) (n-terminal fragment)	PVX_094255	161-1009 (849)	4	WGCF expression
RBP2b (c-terminal fragment)	PVX_094255	1986-2653 (667)	16	WGCF expression
Pv-fam-a	PVX_096995	61-end (420)	6	WGCF expression
MSP8	PVX_097625	24-463 (440)	2.8	WGCF expression

MSP3b	PVX_097680	21-end (996)	1.6	WGCF expression
hypothetical protein	PVX_097715	20-end (431)	6	WGCF expression
MSP3a	PVX_097720	25-end (828)	4	WGCF expression
MSP1-19	PVX_099980	1622-1729 (108)	8	WGCF expression
exported protein	PVX_101530	38-end (330)	2	WGCF expression
Pv-fam-a	PVX_112670	34-end (302)	4.52	WGCF expression
rhoptry neck protein 2 (RON2)	PVX_117880	21-198 (178)	32	WGCF expression
circumsporozoite protein 210 (CSP 210)	PVX_119355	1-348 (349)	4	Expi293
CSP 247	PVX_119355	1-330 (331)	4	Expi293
Pv-fam-a	PVX_125728	30-end (250)	36	WGCF expression

Supplementary Table S2. Antibody seropositivity in the PNG child cohort at week 0. Seropositivity cut-off was defined as the average of negative controls plus two-times the standard deviation. NA= The antibody responses in the children and/or the negative controls were not measured due to antigen unavailability, so seropositivity cannot be determined.

Antigen	IgG	IgG1	IgG3	IgM
AMA 1	36.40%	63.60%	15.20%	0.00%
CSP210	9.10%	69.70%	57.60%	0.00%
CSP247	3.00%	NA	NA	3.00%
CyRPA	NA	NA	NA	6.10%
exported	9.10%	45.50%	9.10%	33.30%
hypothetical protein	9.10%	84.90%	39.40%	6.10%
MSP5	18.20%	33.30%	51.50%	18.20%
MSP7	30.30%	24.20%	36.40%	9.10%
MSP1-19	48.50%	33.30%	36.40%	9.10%
MSP3a	48.50%	63.60%	60.60%	24.20%
MSP3b	42.40%	66.70%	54.60%	15.20%
MSP7B	45.50%	57.60%	42.40%	12.10%
MSP7F	33.30%	42.40%	21.20%	72.70%
MSP7L	51.50%	51.50%	39.40%	48.50%
MSP8	21.20%	72.70%	36.40%	18.20%
PTEX150	39.40%	57.60%	33.30%	12.10%
Pv-fam-a (PVX_088820)	36.40%	51.50%	33.30%	9.10%
Pv-fam-a (PVX_090265)	24.20%	51.50%	51.50%	0.00%
Pv-fam-a (PVX_092990)	6.10%	27.30%	21.20%	12.10%
Pv-fam-a (PVX_096995)	30.30%	75.80%	51.50%	3.00%
Pv-fam-a (PVX_112670)	21.20%	39.40%	42.40%	30.30%
Pv-fam-a (PVX_125728)	21.20%	45.50%	69.70%	6.10%
PVX_090970	15.20%	15.20%	36.40%	0.00%
PVX_091710	24.20%	21.20%	36.40%	12.10%
PVX_101530	54.60%	69.70%	36.40%	75.80%
RAMA	54.60%	60.60%	48.50%	15.20%
$RBP2b_{161-1009}$	NA	97.00%	33.30%	9.10%
$RBP2b_{1986-2653}$	84.90%	93.90%	6.10%	3.00%
RON2	12.10%	30.30%	39.40%	9.10%

s16	15.20%	54.60%	30.30%	9.10%
SIAP2	45.50%	57.60%	39.40%	30.30%
TRAP/SSP2	21.20%	NA	NA	33.30%

Supplementary Table S3. Summary table of epidemiologic data of the Peruvian cohort. The Peruvian cohort study was a longitudinal study performed from December 2012 to December 2015 in Cahuide and San José de Lupuna, Loreto, Peru[3]. Serum samples analyzed in this project were collected at the end of the study period (n=590).

**Epidemiologic characteristics of Peruvian cohort (n=590)**	
**Months of follow-up, n (%)**	
13 months	240 (40.68%)
37 months	350 (59.32%)
**Age, year (range, mean ± SD)**	3.8-85.6 years, 30.1±20.8
<15 y, n (%)	212 (35.93%)
15-39 y, n (%)	192 (32.54%)
≥40 y, n (%)	186 (31.53%)
**Sex, n (%)**	
Male	246 (41.69%)
Female	344 (58.31%)
**Community, n (%)**	
Cahuide	289 (48.98%)
Lupuna	301 (51.02%)
**Total *P. vivax* infections in 13 months by qPCR, n (%)**	
0	146 (24.75)
1-2	287 (48.64)
>=3	157 (26.61)
**Time since last *P. vivax* infection, n (%)**	
Currently infected (<1 month)	160 (27.12)
1-9 months	228 (38.64)
9-13 months	56 (9.49)
None 13 months	75 (12.71)
13-37 months	59 (10.00)
None 37 months	12 (2.03)

Supplementary Table S4. Seropositivity of IgG3 and total IgG in all the Peruvian cohort (n=590) and in those infected within the last 9 months (n=388). Negative control of PVX_094255(RBP2b$_{161-1009}$) was not measured, so the seropositivity cannot be determined. Abbreviation: NA=not available

Antigens	IgG3 seropositivity in cohort, n (%)	IgG3 seropositivity in infected within 9 mo, n (%)	IgG seropositivity in cohort, n (%)	IgG seropositivity in infected within 9 mo, n (%)
AMA 1	206 (34.92%)	159 (40.98%)	463 (78.47%)	313 (80.67%)
CSP210	76 (12.88%)	56 (14.43%)	70 (11.86%)	52 (13.40%)
exported	18 (3.05%)	13 (3.35%)	168 (28.47%)	132 (34.02%)
hypothetical protein	81 (13.73%)	63 (16.24%)	119 (20.17%)	90 (23.20%)
MSP5	243 (41.19%)	189 (48.71%)	241 (40.85%)	190 (48.97%)
MSP7	73 (12.37%)	53 (13.66%)	308 (52.20%)	217 (55.93%)
MSP1-19	149 (25.25%)	108 (27.84%)	481 (81.53%)	315 (81.19%)
MSP3a	188 (31.86%)	138 (35.57%)	285 (48.31%)	219 (56.44%)
MSP3b	82 (13.90%)	65 (16.75%)	478 (81.02%)	321 (82.73%)
MSP7B	200 (33.90%)	148 (38.14%)	542 (91.86%)	358 (92.27%)
MSP7F	48 (8.14%)	39 (10.05%)	65 (11.02%)	54 (13.92%)
MSP7L	106 (17.97%)	77 (19.85%)	252 (42.71%)	177 (45.62%)
MSP8	67 (11.36%)	52 (13.40%)	499 (84.58%)	340 (87.63%)
PTEX150	85 (14.41%)	64 (16.49%)	254 (43.05%)	190 (48.97%)
Pv-fam-a (PVX_088820)	72 (12.20%)	52 (13.40%)	205 (34.75%)	147 (37.89%)
Pv-fam-a (PVX_090265)	35 (5.93%)	30 (7.73%)	436 (73.90%)	301 (77.58%)
Pv-fam-a (PVX_092990)	13 (2.20%)	8 (2.06%)	47 (7.97%)	32 (8.25%)
Pv-fam-a (PVX_096995)	89 (15.08%)	63 (16.24%)	259 (43.90%)	204 (52.58%)
Pv-fam-a (PVX_112670)	49 (8.31%)	35 (9.02%)	254 (43.05%)	192 (49.48%)
Pv-fam-a (PVX_125728)	366 (62.03%)	261 (67.27%)	566 (95.93%)	375 (96.65%)
PVX_090970	46 (7.80%)	30 (7.73%)	466(78.98%)	309 (79.64%)
PVX_091710	23 (3.90%)	16 (4.12%)	476 (80.68%)	317 (81.70%)
PVX_101530	17 (2.88%)	12 (3.09%)	530 (89.83%)	354 (91.24%)
RAMA	26 (4.41%)	18 (4.64%)	187 (31.69%)	140 (36.08%)
RBP2b$_{161-1009}$	42 (7.12%)	29 (7.47%)	NA	NA
RBP2b$_{1986-2653}$	12 (2.03%)	7 (1.80%)	387 (65.59%)	288 (74.23%)
RON2	45 (7.63%)	29 (7.47%)	151 (25.59%)	110 (28.35%)
s16	113 (19.15%)	84 (21.65%)	141 (23.90%)	110 (28.35%)
SIAP2	14 (2.37%)	10 (2.58%)	123 (20.85%)	98 (25.26%)

Supplementary Table S5. Comparison of AUC value of each antigen-specific IgG3 and total IgG in all individuals in Peruvian cohort (n=590) vs in Lupuna only (n=301) in classifying *P. vivax* infections within the last 9 months.

Antigens	IgG3		Total IgG	
	**9 mo (All)**	**9 mo (Lupuna)**	**9 mo (All)**	**9 mo (Lupuna)**
Pv-fam-a (PVX_125728)	0.764	0.864	0.800	0.886
Pv-fam-a (PVX_096995)	0.740	0.779	0.828	0.904
MSP5	0.724	0.813	0.718	0.895
$RBP2b_{1986\text{-}2653}$	0.722	0.787	0.838	0.878
MSP3b	0.714	0.777	0.819	0.893
MSP3a	0.703	0.771	0.835	0.912
PVX_101530	0.686	0.728	0.825	0.895
Pv-fam-a	0.685	0.725	0.824	0.890
MSP1-19	0.676	0.734	0.805	0.873
$RBP2b_{161\text{-}1009}$	0.675	0.738	0.734	0.898
MSP7L	0.673	0.704	0.811	0.892
MSP7B	0.672	0.741	0.835	0.910
CSP210	0.663	0.716	0.584	0.843
AMA1	0.655	0.700	0.805	0.878
hypothetical protein	0.655	0.708	0.651	0.871
PVX_090970	0.654	0.704	0.795	0.882
RAMA	0.652	0.712	0.761	0.873
Pv-fam-a (PVX_092990)	0.648	0.692	0.700	0.830
MSP 8	0.647	0.710	0.820	0.893
PTEX150	0.646	0.691	0.762	0.868
s16	0.639	0.692	0.744	0.898
RON2	0.637	0.694	0.751	0.875
exported	0.634	0.663	0.729	0.854
Pv-fam-a (PVX_088820)	0.625	0.652	0.763	0.886
Pv-fam-a (PVX_112670)	0.623	0.642	0.808	0.896
MSP 7	0.621	0.680	0.777	0.879
PVX_091710	0.617	0.652	0.800	0.879
SIAP2	0.602	0.631	0.757	0.850
MSP7F	0.571	0.589	0.720	0.870
